# An eco‐epidemiological modeling approach to investigate dilution effect in two different tick‐borne pathosystems

**DOI:** 10.1002/eap.2550

**Published:** 2022-03-16

**Authors:** Flavia Occhibove, Kim Kenobi, Martin Swain, Claire Risley

**Affiliations:** ^1^ IBERS, Aberystwyth University Aberystwyth UK; ^2^ UK Centre for Ecology & Hydrology Wallingford UK; ^3^ Department of Mathematics Aberystwyth University Aberystwyth UK

**Keywords:** *Babesia microti*, community assembly, compartmental model, dilution effect, disease ecology, *Ixodes ricinus*, *Ixodes trianguliceps*, Lyme disease, mechanistic model

## Abstract

Disease (re)emergence appears to be driven by biodiversity decline and environmental change. As a result, it is increasingly important to study host–pathogen interactions within the context of their ecology and evolution. The dilution effect is the concept that higher biodiversity decreases pathogen transmission. It has been observed especially in zoonotic vector‐borne pathosystems, yet evidence against it has been found. In particular, it is still debated how the community (dis)assembly assumptions and the degree of generalism of vectors and pathogens affect the direction of the biodiversity–pathogen transmission relationship. The aim of this study was to use empirical data and mechanistic models to investigate dilution mechanisms in two rodent–tick–pathogen systems differing in their vector degree of generalism. A community was assembled to include ecological interactions that expand from purely additive to purely substitutive. Such systems are excellent candidates to analyze the link between vector ecology, community (dis)assembly dynamics, and pathogen transmission. To base our mechanistic models on empirical data, rodent live‐trapping, including tick sampling, was conducted in Wales across two seasons for three consecutive years. We have developed a deterministic single‐vector, multi‐host compartmental model that includes ecological relationships with non‐host species, uniquely integrating theoretical and observational approaches. To describe pathogen transmission across a gradient of community diversity, the model was populated with parameters describing five different scenarios differing in ecological complexity; each based around one of the pathosystems: *Ixodes ricinu*s (generalist tick)–*Borrelia burgdorferi* and *I. trianguliceps* (small mammals specialist tick)–*Babesia microti*. The results suggested that community composition and interspecific dynamics affected pathogen transmission with different dilution outcomes depending on the vector degree of generalism. The model provides evidence that dilution and amplification effects are not mutually exclusive in the same community but depend on vector ecology and the epidemiological output considered (i.e., the “risk” of interest). In our scenarios, more functionally diverse communities resulted in fewer infectious rodents, supporting the dilution effect. In the pathosystem with generalist vector we identified a hump shaped relationship between diversity and infections in hosts, while for that characterized by specialist tick, this relationship was more complex and more dependent upon specific parameter values.

## INTRODUCTION

The dilution effect is the concept that higher biodiversity decreases pathogen transmission in multi‐host/multi‐vector pathosystems as different hosts/vectors present different degrees of transmission competence (Ostfeld & Keesing, 2000; Schmidt & Ostfeld, 2001). This phenomenon has been mainly investigated in the context of zoonotic vector‐borne pathogens, where higher biodiversity seems to reduce human disease risk (LoGiudice et al., 2003; Wood & Lafferty, 2013). Studies focusing on the interactions between pathogen transmission, disease risk and biodiversity have gained progressively more attention due to the current rapid environmental degradation, which seems to drive pathogen eco‐epidemiological dynamics and disease emergence/re‐emergence (Rogalski et al., 2017). Yet, evidence exists that higher diversity increases pathogen prevalence and disease emergence (Dunn et al., 2010; Loaiza et al., 2019), and complex, null, or idiosyncratic relationships between biodiversity and pathogen dynamics have also been observed (Faust et al., 2017; Salkeld et al., 2013). Critiques to the dilution effect regard its generality, the definition of disease risk, spatial scale, and the lack of a mechanistic framework specifically showing the interactions that reduce disease (Strauss et al., 2015).

Ostfeld and Keesing (2000) were among the first to describe the dilution effect, presenting a conceptual model of how Lyme disease risk for humans, in North America, was reduced in regions with higher host species richness and evenness. Lyme disease is caused by *Borrelia burgdorferi* s.l. and is transmitted by an ixodid tick bite to a susceptible host, such as the white‐footed mouse (*Peromyscus leucopus*), the main reservoir of the Ostfeld and Keesing (2000) system. The dilution effect has been empirically observed in more diverse communities, with the underlining assumption that the order in which species assemble is deterministic and positively correlates with their susceptibility, so that more diverse communities will support a relatively larger number of rare species representing low‐competence hosts (LoGiudice et al., 2003; Ostfeld & Keesing, 2000; Schmidt & Ostfeld, 2001). It has been observed in an assembly of nested communities that changes in infection were determined from shifts in host species richness (Lacroix et al., 2014; LoGiudice et al., 2003; Ostfeld & LoGiudice, 2003). Another aspect investigated was the relationship between community richness and the identities or functional traits of its species, with richer communities assumed to have higher functional complexity, and included species that reduced pathogen transmission rates to vectors, or that reduced the abundance of amplification hosts (Johnson & Thieltges, 2010; Pongsiri et al., 2009). This led to the hypothesis that pathogen transmission is likely to rise in disturbed and depleted, communities, especially subjected to anthropogenic stressors (Johnson et al., 2019). However, relationships between wildlife diseases and human‐modified landscapes are rarely consistent, and might depend on pathogen transmission types and landscape scale and features (Brearley et al., 2013).

Nevertheless, the mechanisms underlining dilution were observed to be not directly related to basic biodiversity metrics such as richness or diversity (Johnson, Ostfeld, et al., 2015). For example, in their seminal work in the Lyme disease context, LoGiudice et al. (2008) found that species richness was only weakly negatively correlated with nymphal infection prevalence (NIP), while no relationship existed between the Shannon diversity index and NIP. The relationship biodiversity–pathogen‐prevalence may realistically rely on the identities and frequencies of host species (Strauss et al., 2015). Namely, the dilution effect is connected to how richness scales with relative densities of focal host and non‐host species (Strauss et al., 2015), as well as how nested is the community assembly, and the ecology of pathogen and parasites involved (Wood, Lafferty, et al., 2014). In particular, the direction of the aforementioned relationship depends on the overall community competence (i.e., the sum of each host species’ competence multiplied by its abundance), and how this community competence changes with diversity. If the reservoir hosts abundance increases linearly with richness (additivity), and therefore increases community competence, then high‐diversity systems may support more infection (amplification effect) (Strauss et al., 2015). Alternatively, more diverse communities might include species that reduce reservoir host population sizes (substitution) due, for example, to competition or predation (Wojdak et al., 2014).

Experimental and modeling studies focusing on the dilution effect have traditionally assumed nested communities, which are often observed in nature (Johnson et al., 2012; Wright et al., 1997), and additivity (e.g., Johnson et al., 2013; Roche et al., 2013), although a realistic community might be assembled following a tradeoff between additive and substitutive extremes (Becker et al., 2014; Mihaljevic et al., 2014). It is not likely that total host density increases linearly (i.e., additively) with increasing species richness because the species added to the community might be competitors or predators of reservoir hosts (Levi, Keesing, et al., 2016). So, understanding how the diverse community species abundance differ from less diverse communities is critical in determining how parasite and pathogen transmission is altered when biodiversity is lost (Wojdak et al., 2014).

In vector‐borne pathosystems, such as Lyme disease, it has been thought that more richness has an additive effect on overall tick host density and a reduction in tick infection prevalence as transmission is frequency dependent (more wasted bites on non‐competent hosts; Dobson, 2004; Rudolf & Antonovics, 2005). Yet, adding vector host species could increase overall tick abundance (vector amplification) providing more feeding opportunities to ticks and so a larger number of infected ticks (Randolph & Dobson, 2012; Wood & Lafferty, 2013). In directly transmitted pathogens, such as Hantaviruses, infection probability in the rodent reservoir host decreased when density of sympatric non‐reservoir hosts increased (Khalil et al., 2016; Suzán et al., 2009). In Sweden, the decline of Tengmalm's owls, a main predator of bank voles, which are the PUUV hantavirus reservoir host, has been observed to have contributed to higher PUUV infection rates in that species (Khalil et al., 2016). Likewise, in Africa, the decline of large wildlife increased the landscape‐level prevalence of rodent‐borne diseases (Young et al., 2014). In a mesocosm experiment, diversity of predators was the best negative predictor of trematode infections in frogs (Rohr et al., 2015). In a similar pathosystem, Wojdak et al. (2014) manipulated the density of a focal host (green frog tadpoles), while manipulating the diversity of alternative species, so that alternative species either replaced focal host species thus maintaining the total number of individuals at a constant level (substitution) or added to total host density (addition). They found that parasite transmission remained nearly equal (or decreased slightly) in the first case, while it was higher in case of additivity.

From these examples, it seems that multiple factors, particularly the density of focal host species along the biodiversity gradient and their ecological interactions in the community, regulate the relationship between biodiversity and pathogen transmission, leading to either dilution or amplification effects. Ecological interactions among species might determine the dilution mechanisms originally described by Keesing et al. (2006). These include encounter reduction between reservoir hosts or between hosts and vectors; alteration of host species abundance, distribution, behavior, and overall population dynamics; and competition for resources or space (Civitello & Hartman, 2021; Strauss et al., 2015). Diverse host communities may thus provide two types of dilution hosts: species that are abundant and/or heavily parasitized deflecting contacts between hosts and/or vectors; species that can reduce the abundance of the most competent hosts through interactions such as competition and predation (Levi, Keesing, et al., 2016). However, in vector‐borne pathosystems, for instance those involving generalist ticks, even assuming no additivity, it is plausible that some hosts amplify pathogen transmission because the proportion of “wasted” bites do not compensate the increase in overall tick population due to the potential host species variability in sustaining tick population at different life stages (McCoy et al., 2013).

Hence, another aspect to be considered in the dilution effect debate is the degree of the pathogen/parasite/vector generalism (as opposed as host specialization). Theoretical studies have suggested that the dilution effect will be greatest for pathogens in which within‐species transmission is greater than between‐species transmission and for which transmission is frequency dependent rather than density dependent (e.g., Dobson, 2004; Keesing et al., 2006). Transmission dilution is not expected to occur in the case of highly specialized pathogens and vectors, or when a single host does not allow maintenance of the pathogen cycle, while highly specialized pathogens may facilitate dilution when vectors have a higher degree of generalism (Ostfeld & Keesing, 2012). A recent review suggested that vector‐borne generalist pathogens, especially if transmitted by generalist vectors, were those most likely to be affected by changes to biodiversity (Rohr et al., 2020). Specific studies on ixodid tick species host generalism suggested that generalist patterns certainly vary by tick species but might also be determined by environmental conditions and the scale under consideration, and thus require explicit consideration in epidemiological models of tick‐borne disease (McCoy et al., 2013). However, no mechanistic frameworks integrating realistic community (dis)assembly and the subject of vector degree of generalism exist that are able to investigate the dilution effect mechanisms in a pathosystem, not even in the Lyme disease case, which is among the most studied in the context of the dilution effect.

To consider less extreme and more realistic assembly patterns (Johnson, Ostfeld, et al., 2015), we therefore investigated through a mechanistic eco‐epidemiological approach two tick‐borne pathosystems with different degrees of generalism, and with a community assembly that expanded from purely additive to purely substitutive community structures. We chose two rodent–tick–pathogen systems as wild rodents are ideal wild host communities for studying multi‐host pathogen transmission in the eco‐epidemiological framework, and tick‐borne pathosystems are widely represented in literature allowing parameter estimation and comparison with previous studies. Rodents are abundant and resilient to anthropogenic disturbance (Han et al., 2015; Mendoza et al., 2020). They are reservoirs of numerous zoonotic pathogens of public health interest, such as Lyme disease, Hantavirus diseases, plague, leishmaniasis, various hemorrhagic fevers, human anaplasmosis, and babesiosis (Meerburg et al., 2009). They live in sympatry, sharing pathogens and parasites (e.g., Begon et al., 1999; Paziewska et al., 2012), and displaying a positive correlation between resilience and host reservoir competence (Previtali et al., 2012). Understanding eco‐epidemiological dynamics of rodent‐associated pathogens is crucial from an applied pathogen‐control perspective, as they are predicted to rise as a consequence of large mammal defaunation (Young et al., 2014).

In particular, the two tick‐borne pathosystems chosen were rodent–*Ixodes ricinus*–*Borrelia burgdorferi* s.l. and rodent–*I. trianguliceps–Babesia microti*. Both tick species have a three‐stage life cycle (larva, nymph, and adult). *I. ricinus* (sheep tick) is a generalist tick, of which larvae and nymphs feed on small mammals while adults mostly prefer larger‐sized animals (Mysterud et al., 2015), and it is considered a key vector for *B. burgdorferi* s.l. (the causative agent of Lyme disease in humans; Paziewska et al., 2010). *I. trianguliceps* is a specialist tick for small mammals (Mysterud et al., 2015), which, in the UK, has been found to be the key vector for *B. microti*, a potentially zoonotic protozoan, of which voles seem to be the main reservoir (Bown et al., 2008; Hussein, 1980; Siński et al., 2006). These two pathogens have also different infectious periods (Harrison et al., 2011; Hartemink et al., 2008; Randolph, 1995; Randolph et al., 1996). *I. ricinus* is sympatric with *I. trianguliceps* in some areas, but the role of the former in *Babesia* sp. transmission, and the zoonotic potential of the latter are still unclear (Bown et al., 2006; Kovalevskii et al., 2013).

We integrated unique empirical data on wild rodent communities and mechanistic eco‐epidemiological modeling to improve our understanding of the functional form of density dependence for the tick population, particularly with regard to the density of infectious nymphs (DIN), and how the presence of predators and competitors influences the density of amplification hosts. Ground‐dwelling rodent species were surveyed in Wales (UK) in order to collect ad hoc empirical data to parametrize a compartmental model including epidemiological and population dynamics of host and vector, as well as ecological relationships such as competition and predation, with species able to affect host and vector populations but incapable of maintaining pathogen transmission. Simulations of realistic, nonrandom community assemblages were performed to investigate how the interactions between host and non‐host species affected pathogen dynamics and the mechanisms behind the potential dilution or amplification effect. Our simulations aimed to identify (1) whether the dilution and amplification effects occurred under the assumption of partially additive host densities and which were the mechanisms involved; (2) whether these effects occurred in both pathosystems and whether there were differences due to the different degree of vector generalism; and (3) whether there were identifiable specific characteristics in each pathosystem determining dilution or amplification to provide practical disease risk management recommendations (i.e., if there were crucial information on the community or the vector in a specific area to be collected to make realistic predictions on potential disease risk). These were particularly relevant to the study area, where rodent tick‐borne zoonotic risk was not previously assessed despite the close interface between human‐livestock‐wildlife. The additional knowledge provided on tick‐borne pathosystem dynamics fits into a growing corpus of information required to improve wildlife management and zoonotic risk control policies in these unprecedented times of biodiversity decline.

## METHODS

### Ground‐dwelling rodent populations survey

Rodents were live trapped twice a year (spring and autumn) in seven different sites in Wales between June 2015 and June 2017, including a range of different habitats (semi‐deciduous woodland, conifer plantation, grassland, clear‐cut/scrubland). At each location, the square grid consisted of 36 trapping stations (6 × 6) with one Longworth and one Sherman trap at each station to maximize estimation of species composition (Anthony & Ribic, 2005). The traps were set with appropriate bedding material and food; each trapping occasion consisted of four consecutive days and nights, with the first day/night being pre‐baiting, and the traps were checked twice a day (early morning and sunset). The live‐trapping was performed during two different seasons to estimate individual densities of the pre‐breeding recruitment population (May–June) and the post‐breeding peak population (September–October). Each individual, at the time of first capture, was identified at the species level, sexed, assigned to an age class according to Telfer et al. (2002), weighed, individually marked by fur clipping, and finally released. On first capture only, ticks were collected from each individual, and stored for further molecular investigations (Occhibove, 2018).

Individual density was estimated by species, site, and trapping season using the POPAN algorithm (Schwarz & Arnason, 1996) within the software MARK (White & Burnham, 1999), assuming an open population, constant survival, and constant capture probability. Goodness‐of‐fit was tested with the RELEASE suite within the software. Growth rate, *r*, was estimated for each species according to the formula proposed by Lambin et al. (2000): *r*_*i*_ *=* log*N*_*i*(*t*)_ − log*N*_*i*(*t*−1)_ where *N*_*i*(*t*)_ is the population density of species *i* at time *t*. This process generated distinct growth rates representing breeding and non‐breeding seasons, March to October and November to February respectively according to Hörnfeldt (1994) and Stenseth et al. (2002). Unless specified differently, all data analyses were performed in R (R Core Team, 2016).

### Model structure and simulations

**FIGURE 1:**
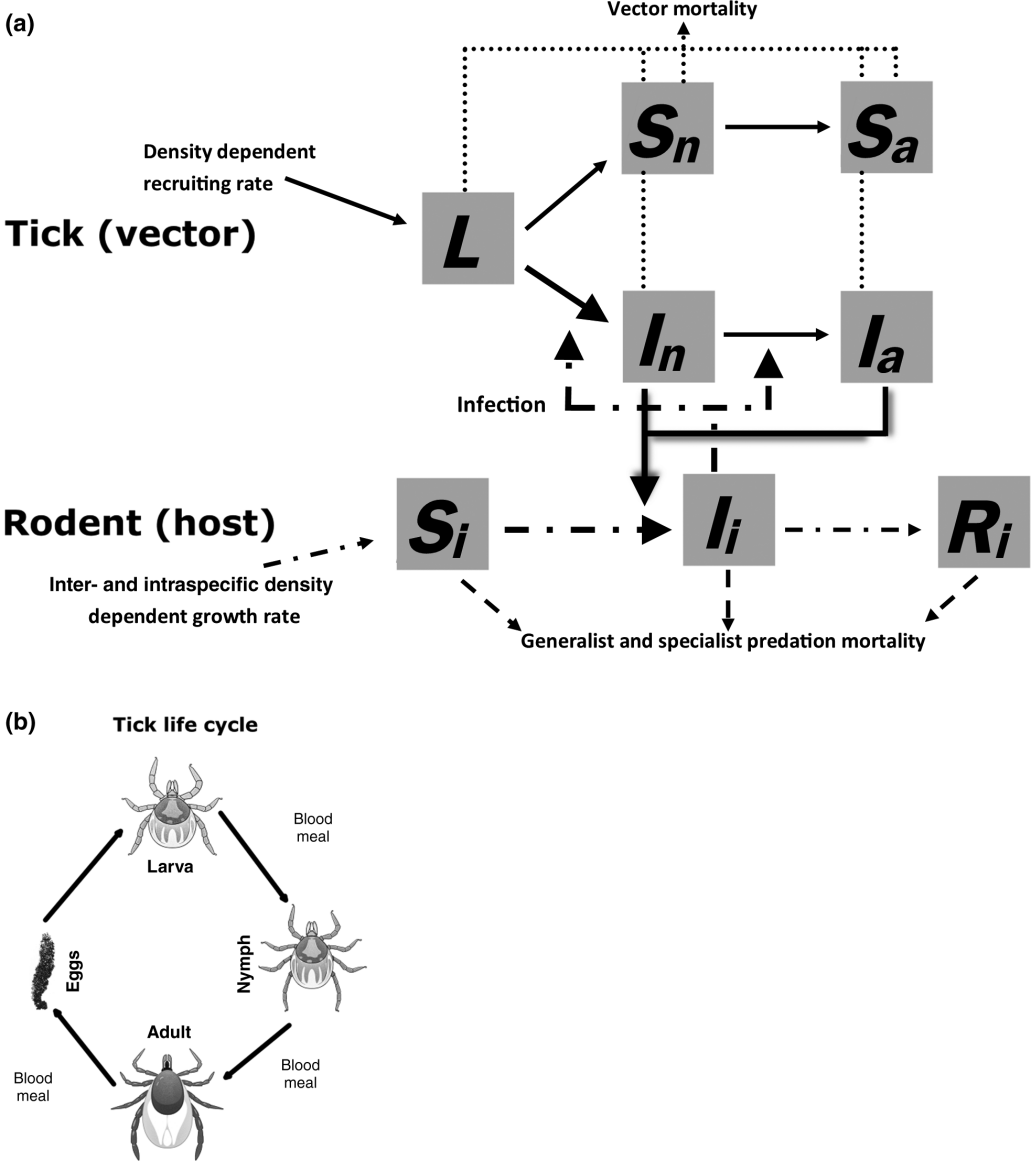
(a) Tick‐borne disease compartmental model and (b) tick life cycle. Boxes represent epidemiological compartments in which each population is subdivided: *L*, tick larval stage; *S*, susceptible; *I*, infectious; *R*, recovered. Subscripts are *n*, tick nymphal stage; *a*, tick adult stage; *i*, rodent species (host). Vectors can feed also on non‐host competitor species (shrew) and specialist predators. Arrows indicate the direction of movement of individuals between classes (solid line, vector; dash‐dotted line, host). Arrows pointing outside the boxes represent mortality (dashed lines, host mortality through predation; dotted lines, vector natural mortality). Thicker arrows represent transmission. Each of the five scenarios in Table 1 were based on this model, with non‐host competitor occurring in scenarios 3 to 5, generalist and specialist predation occurring only in scenarios 4 and 5

Similarly to Arino et al. (2004) and Venturino (1994, 2001, 2002), we developed a demographic model that accounted for interactions between different populations in which the pathogen spreads among host species but does not affect their survival rates. Indeed, *B. burgdorferi* s.l. and *B. microti* have not been observed to impact rodent mortality (Bown et al., 2008; Randolph et al., 1996; Telfer et al., 2008; Voordouw et al., 2015). Our modeling approach consisted of a deterministic multi‐host single‐vector SIR (Susceptible‐Infectious‐Recovered)‐SI (Susceptible‐Infectious) compartmental model made up of a set of differential equations (Appendix S1: Equations S1–S18 and model code in Data S1: Tick_borne_model.R). Figure 1 illustrates the structure of the model (Figure 1a) and the tick life cycle (Figure 1b). All the results were produced using the function rk4 in the R package *deSolve* (R Core Team, 2016), which is based on the classical Runge–Kutta fourth‐order integration. The model was not explicitly spatial. The area, 1 ha, was considered constant since it was constrained by the experimental sampling unit, therefore the populations were expressed in individuals/ha and the relevant parameters were scaled accordingly. Dilution was tested by modeling a progressively more complex community: species were added in turn following nonrandom assembly rules. The structure of the model was chosen to offer a compromise between complexity and parsimony, and it was parametrized, where possible, with data collected during live trapping.

Five scenarios (Table 1) for the community assembly were simulated based around the structure outlined in Figure 1, with each scenario representing an increase in complexity from the previous one:

1. Scenarios 1 and 2: as was found in field surveys, wood mice and bank voles were rodent host species for both the pathogen and the tick vectors. Scenario 1 involved one host species (bank vole), whereas Scenario 2 involved both wood mice and bank voles.
2. Scenario 3: *Sorex* ssp. shrews were added as a sympatric competitor species not hosting the pathogen. Evidence of their presence (shrew feces found in traps equipped with shrew escape holes) were found in the sampling sites, and they were suitable hosts for ticks.
3. Scenario 4: addition of two types of predators, specialists and generalists. Specialist predators may be represented by mustelids and are also suitable tick hosts. Generalist predators may be represented by birds and do not affect tick population.
4. Scenario 5: addition of a large grazing ungulate, i.e., sheep or deer, which is a suitable host for adult stages of *I. ricinus* but incapable of pathogen transmission. Otherwise ungulates are assumed to be a constant population not affecting, or affected by, any population in the system.

Each of these five scenarios were simulated for both pathosystems (*I. ricinus‐B. burgdorferi* and *I. trianguliceps‐B. microti*) giving 10 different modeled scenarios in total. Although Scenario 5 in the second pathosystem was identical to Scenario 4 due to the tick specialization on small hosts and the null effect of ungulates on other species population dynamics.

**TABLE 1:** Species composition for each community assemblage scenario

Name	Community composition^a^	Species role in the community			Vector life stage preferentially hosted	
					*I. ricinus* (generalist tick)	*I. trianguliceps* (specialist tick)
Scenario 1 (single host)	Bank vole	Pathogen host	Vector host	Rodent competitor	Larva, nymph	Larva, nymph, adult
Scenario 2 (two hosts)	Wood mouse	Pathogen host	Vector host	Rodent competitor	Larva, nymph	Larva, nymph, adult
Scenario 3 (rodents + competitor)	*Sorex* sp. shrew		Vector host	Rodent competitor	Larva, nymph	Larva, nymph, adult
Scenario 4 (Rodents + competitor + predation)	Specialist predator (mustelid)		Vector host	Predator of all species	Nymph, adult	…
	Generalist predator			Predator of all species	…	…
Scenario 5 (full community)	Ungulate (e.g., sheep or deer)		Vector host		Adult	…

*Note*: The communities are nested in that each sequential scenario includes the species of previous scenarios in addition to the additional species listed. Each of these five scenarios was simulated for each of the two pathosystems (*Ixodes ricinus–Babesia burgdorferi* and *Ixodes trianguliceps–Babesia microti*).

^a^ Each scenario incorporates each of the species in rows above in addition to those in the scenario row.

Rodent and shrew populations were modeled according to the Lotka‐Volterra system, namely a logistic growth tending to a species‐specific carrying capacity and limited by intra‐specific density dependent reduction and interspecific competition (Hanski et al., 1993; Lotka, 1925), as these are considered crucial population dynamics in rodents (Burthe et al., 2010; Occhibove, 2018; Smith et al., 2006). Interspecific competition among rodents, and between rodents and shrews was modeled with a density‐dependent competition term (Huitu et al., 2004; O'Regan et al., 2015), considering rodent species better competitors than shrews (Henttonen et al., 1989; Huitu et al., 2004). These species were all predated upon, but rodent species were considered preferential prey (Korpimäki, 1992; Korpimäki & Norrdahl, 1989). Generalist predation was modeled according to the alternative prey hypothesis (Holling type III functional response), while specialist predator population was modeled according to the Holling type II functional response based on the Rosenzweig‐MacArthur model (1963) with no preference among rodents (Erlinge, 1975; Hanski & Henttonen, 1996; Krebs & Myers, 1974). Specialist predation parameters were based on *Mustela nivalis* (least weasel; Table 2), which was widespread across sampled sites. Tick population was modeled with a density‐dependent fecundity reduction term (Norman et al., 1999; Ogden et al., 2007) and aggregation, i.e., non‐homogeneous distribution of vectors among the host population (Rosà et al., 2003). The aggregation term was parametrized according to the results of tick collection during the rodent survey (reported in *Parameter estimation*; Table 2).

**TABLE 2:** State variable initial values and parameter values (range of values included in sensitivity analysis are included in brackets)

Symbol	Description	Value	Source
*N*_w_	Wood mouse population (initial)	49	This study
*N*_b_	Bank vole population (initial)	75	This study
*N*_j_	Shrew population (initial)	20	This study
*N*_p_	Weasel population (initial)	3	This study
*N*_d_	Ungulate population (throughout)	5	This study
*N*_v_	Tick population (initial)	100	This study
cbw, cjw	Competition of wood mouse over bank vole, and shrew respectively	0.20 1.04	This study based on O'Regan et al. (2015)
cwb, cjb	Competition of bank vole over wood mouse, and shrew respectively	0.20 1.03	This study based on O'Regan et al. (2015)
cwj, cbj	Competition of shrew over wood mouse, and bank vole respectively	0.11 0.12	This study based on O'Regan et al. (2015)
*d*_r_ *d*_s_ *d*_l_	Molting‐feeding success on Rodents Shrews Larger hosts (weasel/ungulate)	0.14 (0.1–0.59) 0.16 (0.1–0.50) 0.21 (0.1–0.64)	LoGiudice et al. (2003)
*G*	Saturation rate of generalist predation	0.49	This study based on Turchin & Hanski (1997)
*H*	Prey density at which generalist predation rate is half of the maximum	9.9	This study based on Turchin & Hanski (1997)
*K*	Tick aggregation parameter	0.18	This study based on Rosà et al. (2003)
num_egg_	Maximum number of per capita adult female tick eggs production *Ixodes ricinus* *I. trianguliceps*	1500 1000	*I. ricinus*: Norman et al. (1999, *I. trianguliceps*: Krasnov et al. (2007)
*q*	Specialist predator–prey ratio constant	56	This study based on Turchin & Hanski (1997)
*r*_b*+*_, *r*_b*−*_	Bank vole growth rate breeding season (+), and non‐breeding season (−)	0.007; −0.002	this study
*r*_w*+*_, *r*_w*−*_	Wood mouse growth rate breeding season (+); non‐breeding season (−)	0.04; −0.006	this study
*s*_v_	Density‐dependent reduction of tick growth rate	‐	Formula in Ogden et al. (2007)
Α	Maximum rodent consumption rate of specialist predator	1	This study based on Turchin & Hanski (1997)
α_s_	Maximum shrew consumption rate of specialist predator	7.67	This study based on Turchin & Hanski (1997)
β_sl_ β_sn_ β_sa_	Encounter rate small host/larva *I. ricinus; I. trianguliceps* Encounter rate small host/nymph *I. ricinus; I. trianguliceps* Encounter rate small host/adult *I. ricinus; I. trianguliceps*	0.040; 0.040 0.040; 0.040 0.020; 0.040	Dobson et al. (2011), Hancock et al. (2011)
β_ll_ β_ln_ β_la_	Encounter rate large host/larva *I. ricinus; I. trianguliceps* Encounter rate large host/nymph *I. ricinus; I. trianguliceps* Encounter rate large host/adult *I. ricinus; I. trianguliceps*	0.025; 0.000 0.040; 0.000 0.060; 0.000	Dobson et al. (2011), Hancock et al. (2011)
Δ	Half‐saturation constant (rodent)	11.31	This study based on Turchin & Hanski (1997)
Δ_s_	Half‐saturation constant (shrew)	22.62	This study based on Turchin & Hanski (1997)
ν_j_	Shrew birth rate	2.60	This study based on de Leo & Dobson (1996), Bolzoni et al. (2008)
ν_p_	Weasel birth rate	3.23	This study based on de Leo & Dobson (1996), Bolzoni et al. (2008)
ρ_j_	Shrew death rate	1.04	This study based on de Leo & Dobson (1996), Bolzoni et al. (2008)
ρ_p_	Weasel death rate	1.29	This study based on de Leo & Dobson, 1996, Bolzoni et al., 2008)
ρ_v_	Tick death rate: larva nymphs adults	0.0014 0.0005 0.0004	Dobson et al. (2011)
σ_bb_	Recovery rate *Borrelia burgdorferi* s.l.	0.0083	Harrison et al. (2011), Hartemink et al. (2008), Randolph (1995), Randolph et al. (1996)
σ_bm_	Recovery rate *Babesia microti*	0.4	Harrison et al. (2011), Hartemink et al. (2008), Randolph (1995), Randolph et al. (1996)
τ	Reservoir competence of transmission: Host to vector Vector to host	0.50 (0.1–1) 0.80 (0.1–1)	Giardina et al. (2000), Harrison et al. (2011), Hartemink et al. (2008), LoGiudice et al. (2003)

*Note*: Rates are expressed per day in accord with the model time step.

A simulation time period of 20 years, with a daily time step, allowed equilibration. The model included susceptible, infectious, and recovered compartments (SIR) for each competent mammalian host; and susceptible and infectious (SI) for the vector (Porco, 1999). Non‐systemic transmission through tick co‐feeding was not considered because it was found to be a very minor, or inefficient route of transmission for the chosen pathogen (Jacquet et al., 2016). Both pathogens under consideration do not present vertical transmission, consequently larvae were not infectious, but could be infectious and molt into infectious nymphs (Gray, 2006; Randolph, 1995). Reservoir competence values summarized susceptibility, ability of the pathogen to magnify and persist in the host/vector and efficiency of transmission; all of the individuals of the same host species were equally competent, but different species might display different levels of competence. Initial density for each rodent species was set at the average value found in the field (bank vole, 75 ± 68 individuals/ha; wood mouse, 49 ± 53 individuals/ha). The inoculum was represented by a single infectious individual. Appendix S1: Equations S1–S7 represent the model of competent, non‐competent host species and vector dynamics in the absence of pathogen, while Appendix S1: Equations S8–S18 represent the SIR‐SI dynamics when the pathogen is introduced. Model variables and parameters are listed in Table 2 together with initial conditions for simulations and parameter values. Model code provided in Data S1.

The model outputs of interest were number of infectious hosts and number of infectious nymphs, named hereafter density of infectious nymphs (DIN; from Giardina et al., 2000). These were chosen because they are commonly used to estimate disease risk (LoGiudice et al., 2003; Piesman, 1989); in particular DIN is considered a better metric than nymphal prevalence to assess human zoonotic risk (LoGiudice et al., 2008). However, more relevance will be given to the actual numbers of infectious individuals as they provide the best interpretation of transmission dynamics without interference of effects from population dynamics across different communities (i.e., change in susceptible host population due to community assembly that altered the infectious proportion).

### Model parameterization

Parameters were estimated, where possible, from rodent live‐trapping in this study, otherwise they were taken from relevant literature (Table 2 and Appendix S2). Rodent species growth rates (*r*) in the model were the averages for each species and for season (Appendix S3: Table S1) scaled by day. Seasonal growth rates (*r*) were used to simulate rodent intra‐annual fluctuations and the tick population diapause (Randolph, 2004).

Carrying capacity (*K*), and birth and death rates of non‐host species were estimated allometrically (Bolzoni et al., 2008; de Leo & Dobson, 1996; Appendix S2). The competition factor (*c*) was computed algebraically (Appendix S2). For the simulations, we used conservative values of competition (Table 2), according to Occhibove (2018). Predation parameters were estimated with methods in Turchin and Hanski (1997), separately for rodents and shrews, as the latter have a significantly lower body mass and are secondary prey items (Henttonen et al., 1989).

Parameters regarding ticks with relative source and value are listed in Table 2. Only the aggregation parameter (*k*) was calculated from empirical data (Appendix S2), while other parameters were taken from relevant literature allowing to model the differences in degree of generalism and ecology in general among the two tick species. Values for reservoir competence of hosts and vector, host–vector encounter rates, and molting success were taken from literature (Table 2). Recovery rates (σ) were pathogen specific and represented the reciprocal of the infectious period (Table 2).

### Sensitivity analysis

By keeping constant the parameters estimated in this study and parameters characterizing the differences of the two pathosystems, a sensitivity analysis was performed on parameters that were not empirically estimated. The ranges for these parameters were selected from various studies on the most similar pathosystems, namely molting success on various hosts, and reservoir competence. These parameters were observed to greatly affect disease transmission and dilution output (Dunn et al., 2013; Roche et al., 2013), but uncertainty on the values existed for the pathosystems under consideration. We used Latin Hypercube Sampling (LHS) and partial rank correlation coefficients (PRCC) to assess the impact of uncertainty and the sensitivity of the outcomes of the simulations to variations in each parameter (Iman et al., 1981a, 1981b; Marino et al., 2008). To generate the LHS matrices, we assumed that all the selected parameters were uniformly distributed. Then a total of 1000 simulations of the models per scenario were carried out. The baseline conditions and the ranges used are given in Table 2; the ranges were chosen as minimum and maximum values found from empirical studies in literature for each parameter. Sensitivity analysis was performed separately for each host–tick–pathogen system, keeping the parameters not subject to sensitivity analysis constant. The response functions used were the density of infectious nymphs (DIN) and the number of infectious hosts at equilibrium.

## RESULTS

### Ground‐dwelling rodent populations survey

During the 4968 trap‐nights of fieldwork, 258 bank voles (*Myodes glareolus*) and 230 wood mice (*Apodemus sylvaticus*) were captured, with signs of shrew visits in every trapping grid, i.e., shrew feces were recovered in trap tunnels. Sex ratios were balanced with a 1.08 males per female overall, while the ratio of wood mice to bank voles was 0.86. Inter‐seasonal fluctuations of population density were evident, and, as expected, higher density was found in the post‐breeding‐peak season (autumn). Non‐breeding season growth rates were mostly negative, or much lower than breeding season values in both species (Appendix S3: Table S1). General trends were similar between the two species. Density values across all field seasons were much lower than the estimated carrying capacity for each species, suggesting that in our sites the populations might be subjected to interspecific competition and predation. Hence, model structure reflected field observations; numerical simulations for rodent population sizes across different scenarios are shown in Appendix S3: Figure S1.

Ticks were found to be highly aggregated on hosts and the dispersion parameter of the negative binomial distribution fitting field data was used in the model to represent aggregation (*k* was 0.18, indicating a much more aggregated distribution that is frequently observed in ticks; see, e.g., Ferreri et al., 2014). In total, 225 ixodid ticks were collected from 120 rodents, 16.28% of total individuals sampled. Total infestation prevalence (i.e., including all occasions rodents were caught irrespective of previous trapping status) was 15.99%, with *I. trianguliceps* representing most of the sample set (196 specimens), followed by *I. ricinus* (10 specimens), while 19 specimens were not taxonomically identifiable.

### Sensitivity analysis

Results of the sensitivity analysis showed that the parameters overall mostly influencing the outputs in both pathosystems were molting success on rodents, and transmission competence vector to rodent (Appendix S3: Figure S3). As these parameters made a substantial impact on the conclusions drawn from the main modeling results, results of model simulations were displayed with respect to these two influential parameters.

### Model simulations

Firstly, host–vector interactions were investigated through simulations in the absence of pathogens to display the differences in dynamics of the two tick species. Literature indicated that fecundity of *I. ricinus*, is great er than *I. trianguliceps*. The inclusion of this difference in the no‐pathogen model resulted in a higher number of individuals across all scenarios for the generalist tick *I. ricinus* when compared to *I. trianguliceps*; more so when larger hosts were added to the community (Scenario 4/5; Appendix S3: Figure S2). Conversely, *I. trianguliceps* only feeds on small hosts (rodents and shrews) therefore there was no substantial change in tick numbers across different communities, apart from Scenario 3, when the shrew population was added, determining an overall host increase and consequent vector increase (Appendix S3: Figure S2). Tick populations were subjected to a density dependent fecundity reduction, therefore did not linearly increase with host population.

**FIGURE 2:**
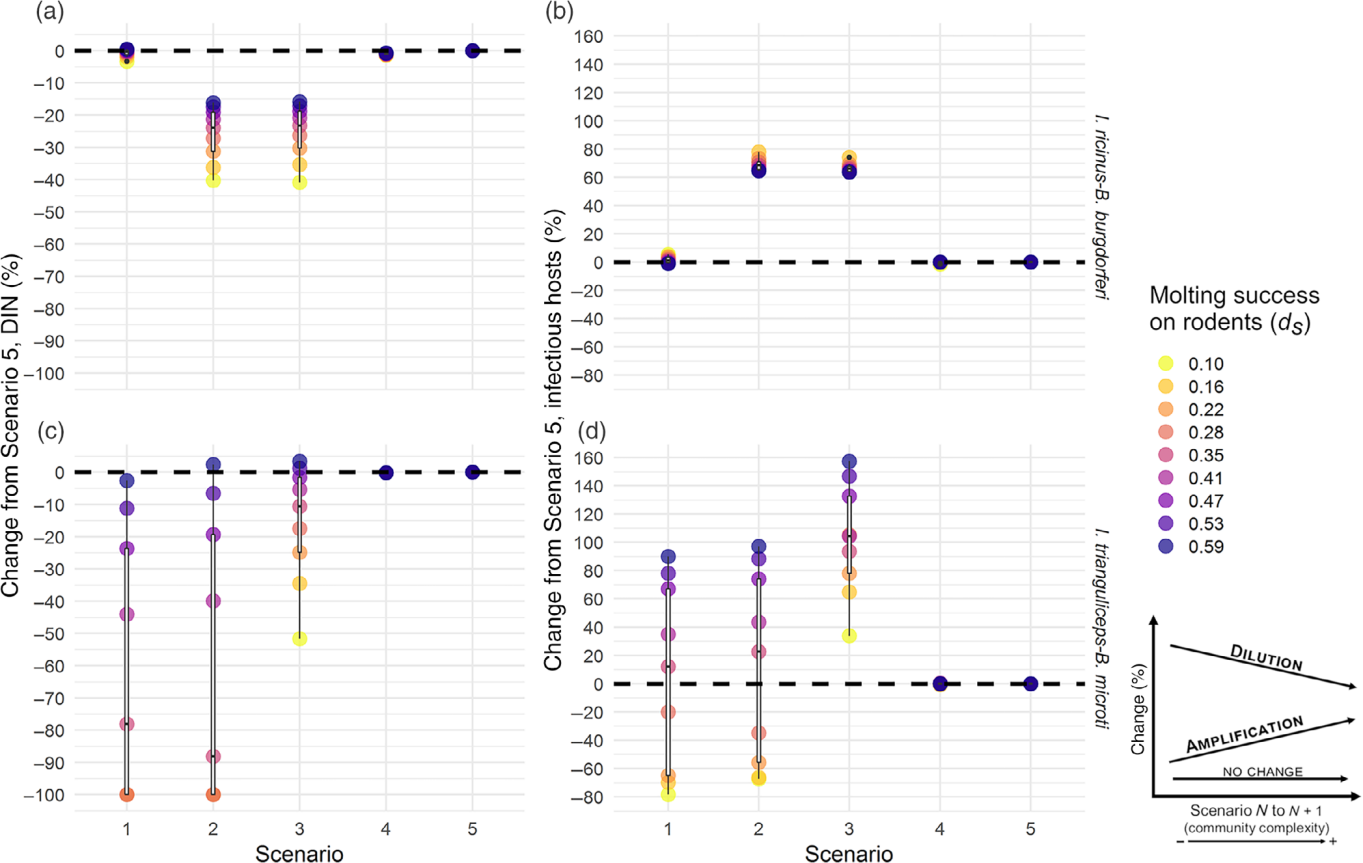
Percentage of change from Scenario 5 in (a, b) density of infectious nymphs (DIN) and (c, d) infectious hosts across the five scenarios (on the *x*‐axis) and the range of molting success on rodents (*d*_*s*_) (colored circles) in the two pathosystems: *Ixodes ricinus–Babesia burgdorferi* (top) and *I. trianguliceps–B. microti* (bottom). Black dashed line, no change. Box and whisker plots on top of colored circles represent the percentage of change distribution across the range of *d*_*s*_ values. Lower and upper box boundaries are 25th and 75th percentiles, respectively, line inside box is the median, lower and upper error lines are 10th and 90th percentiles, respectively, filled circles show data falling outside 10th and 90th percentiles. Decrease of percentage of change from less complex to more complex scenario represents dilution, while increase represent amplification. Other parameter values are listed in Table 2

**FIGURE 3:**
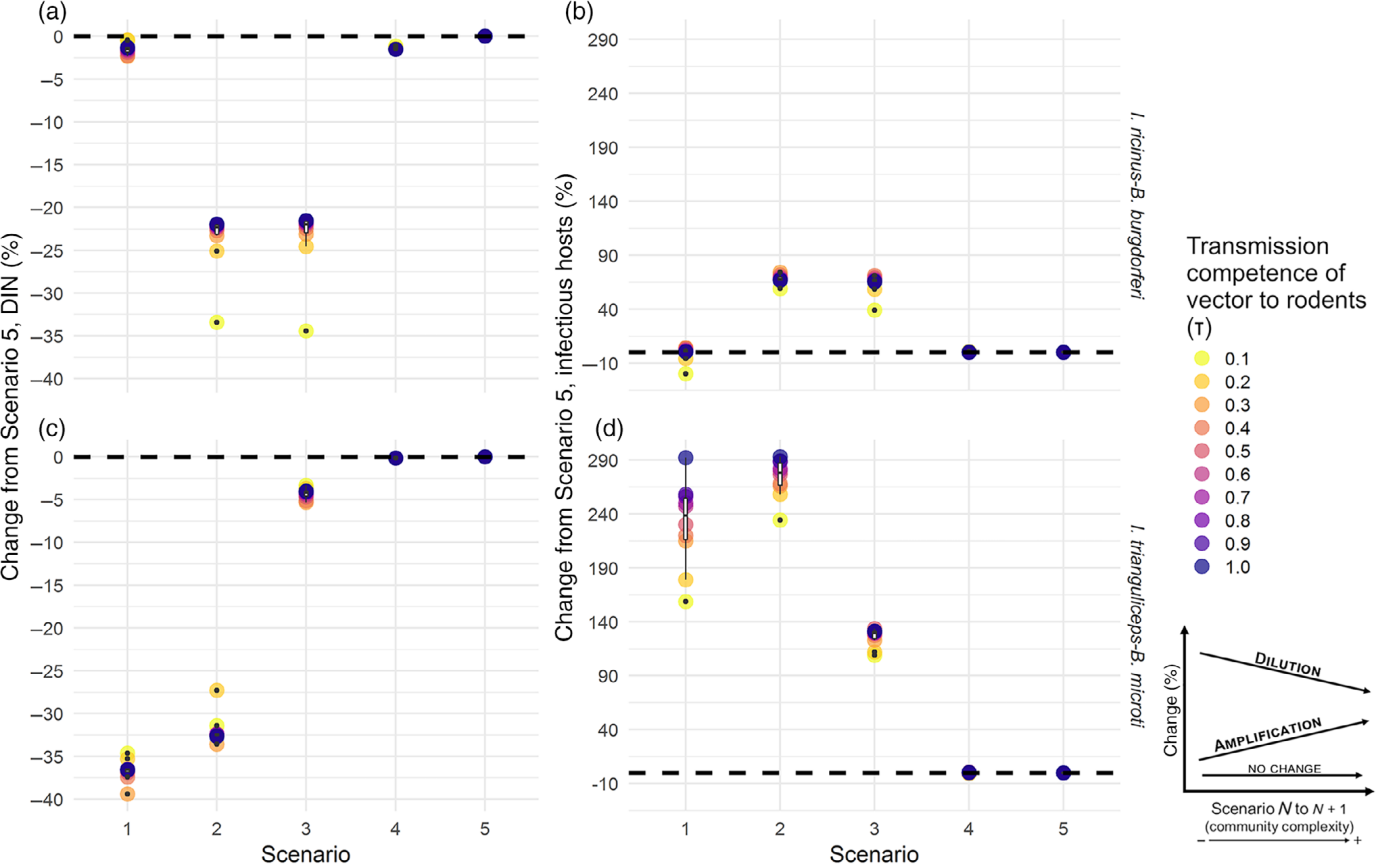
Percentage of change from Scenario 5 in (a, b) DIN and (c, d) infectious hosts across the five scenarios (on the *x*‐axis) and the range of transmission competence of vector to rodents (*τ*, colored circles) in the two pathosystems: *I. ricinus–B. burgdorferi* (top) and *I. trianguliceps–B. microti* (bottom). Black dashed line, no change. Box and whisker plots on top of colored circles represent the percentage of change distribution across the range of τ values. Lower and upper box boundaries are 25th and 75th percentiles, respectively, line inside box is the median, lower and upper error lines are 10th and 90th percentiles, respectively, filled circles show data falling outside 10th and 90th percentiles. Decrease of percentage of change from less complex to more complex scenario represents dilution, while increase represent amplification. Other parameter values are listed in Table 2

As our sensitivity analysis showed that molting success on rodents (henceforth referred to as simply molting success), and the competence of transmission from vector to rodent affected model output the most (see previous section), Figures 2 and 3 display model output variation across scenarios over the range selected for those parameters (i.e., the percentage change in the output of interest taken at equilibrium). In the *I. trianguliceps*–*B. microti* system, Scenario 5 was identical to Scenario 4 due to the specialization of the vector for small hosts and the absence of population interactions between ungulates and other hosts.

As expected, in the *I. ricinus*–*B. burgdorferi* system, DIN increased together with the increase of the molting success, while the opposite trend could be observed for prevalence because of the rise of total tick population (not shown). Single‐host scenario (Scenario 1) and the most complex communities (Scenario 4 and 5) gave virtually identical results (at equilibrium), with the only difference being a slightly lower DIN in the first case at low molting success values (Figure 2a). On the other hand, Scenario 2 and 3 resulted in a lower DIN compared to the other communities across the entire range of parameter values and displayed very similar values. The DIN difference between the two groups of scenarios (Scenario 1, 4 and 5 vs. Scenario 2 and 3) decreased while increasing the parameter value; yet, at the highest end of the range, DIN was still higher ~25% in the simplest community (large population of competent host) and in the communities where tick population was amplified by a larger number of hosts (Figure 2a). This means that we could observe a dilution effect with the introduction of competitor species, but this was outweighed in Scenario 4 and 5 by the introduction of other host species for ticks. Changing the value of molting success only made very slight differences to the total number of infectious hosts. However, Scenario 2 and 3 (which gave almost identical results in this case) presented the highest number of infectious hosts; while the increase in community complexity in Scenario 4 and 5 resulted in a reduction of transmission at levels equal to or even lower than Scenario 1 (at low molting success values), producing dilution of transmission (Figure 2b).

Similarly, the increase of molting success determined an increase in DIN and infectious hosts in the second pathosystem, *I. trianguliceps*‐*B. microti*, although there were major differences between the two systems. DIN increase over the range of the parameter was significant, with all scenarios reaching similar numbers of infectious nymphs at the highest end of the molting success value (Figure 2c). In this case, scenarios are roughly ordered, in terms of DIN, from the least complex having lower values of DIN to the most complex. Namely, the increase in small‐sized hosts, for which this tick is a specialist, determined a DIN increase. Yet, the introduction of predation in Scenario 4, which determined a significant reduction of competent hosts but not shrews, resulted in slightly lower DIN. This is because the tick population was sustained by the shrew population, but it was slightly less likely for tick individuals to get infected on the less infectious hosts (Figure 2b). Therefore, only little dilution effect could be observed. Nonetheless, dilution could be identified in hosts, for any value of molting success, when predation was introduced (Scenario 4) with respect to Scenario 3. This occurred only above certain molting success values in the case of the first two scenarios (Figure 2d).

When varying the competence of transmission from vector to rodent parameter a very similar pattern compared to the previous case could be observed in the *I. ricinus*–*B. burgdorferi* system. The variation in the parameter had relatively little effect on the DIN in the same scenario. However, it decreased from Scenario 1 to 2, then did not significantly change with the introduction of shrews (Scenario 3), while it increased due to the introduction of predators and ungulates (Scenario 4 and 5; Figure 3a). This might have been the result of an interaction between the relaxation of the fecundity reduction with consequent tick release (less small mammals in Scenario 4 and 5), and the modeled aggregation on competent hosts (higher probability of finding an infectious host). Conversely, infectious hosts increased when the second host species was added to the community (Scenario 2), remained stable when shrew population was added (excluding a very low competence value; Scenario 3), while decreasing in the most complex communities simulated (Scenario 4 and 5), which presented the same results (Figure 3b). Nevertheless, at very low competence values, the single‐host community (Scenario 1) presented the least infectious hosts. DIN values and the number of infectious hosts did not increase linearly with competence as the tick population was regulated by a fecundity reduction function and was unevenly distributed in the host population (see *Results: Ground‐dwelling rodent populations survey*) and therefore no infinite increase of infections was possible. In addition, *I. ricinus* reproduced abundantly and being generalist had an abundance of hosts in every scenario, so low competence values were sufficient to sustain transmission.

The competence of transmission from vector to rodent appeared to be crucial in determining the final number of infectious hosts in the second pathosystem (*I. trianguliceps*–*B. microti*) when only rodents were in the community (Scenario 1 and 2; Figure 3d), while relatively no significant change could be observed across the parameter range for DIN in the same scenario (Figure 3c). Namely, a small level of competence was enough to saturate transmission in nymphs due to tick specialization, while an increase in competence of transmission from vector to rodent increased the number of infectious hosts in Scenarios 1–3. In this pathosystem, no dilution was observed when DIN was taken as metric of interest; on the contrary, the more hosts and the more complex the community, the more transmission seemed to be amplified (Figure 3c). However, shrews (Scenario 3) and predation (Scenario 4) determined a reduction of infectious hosts compared to the only rodents scenarios (Scenarios 1 and 2; Figure 3d).

**FIGURE 4:**
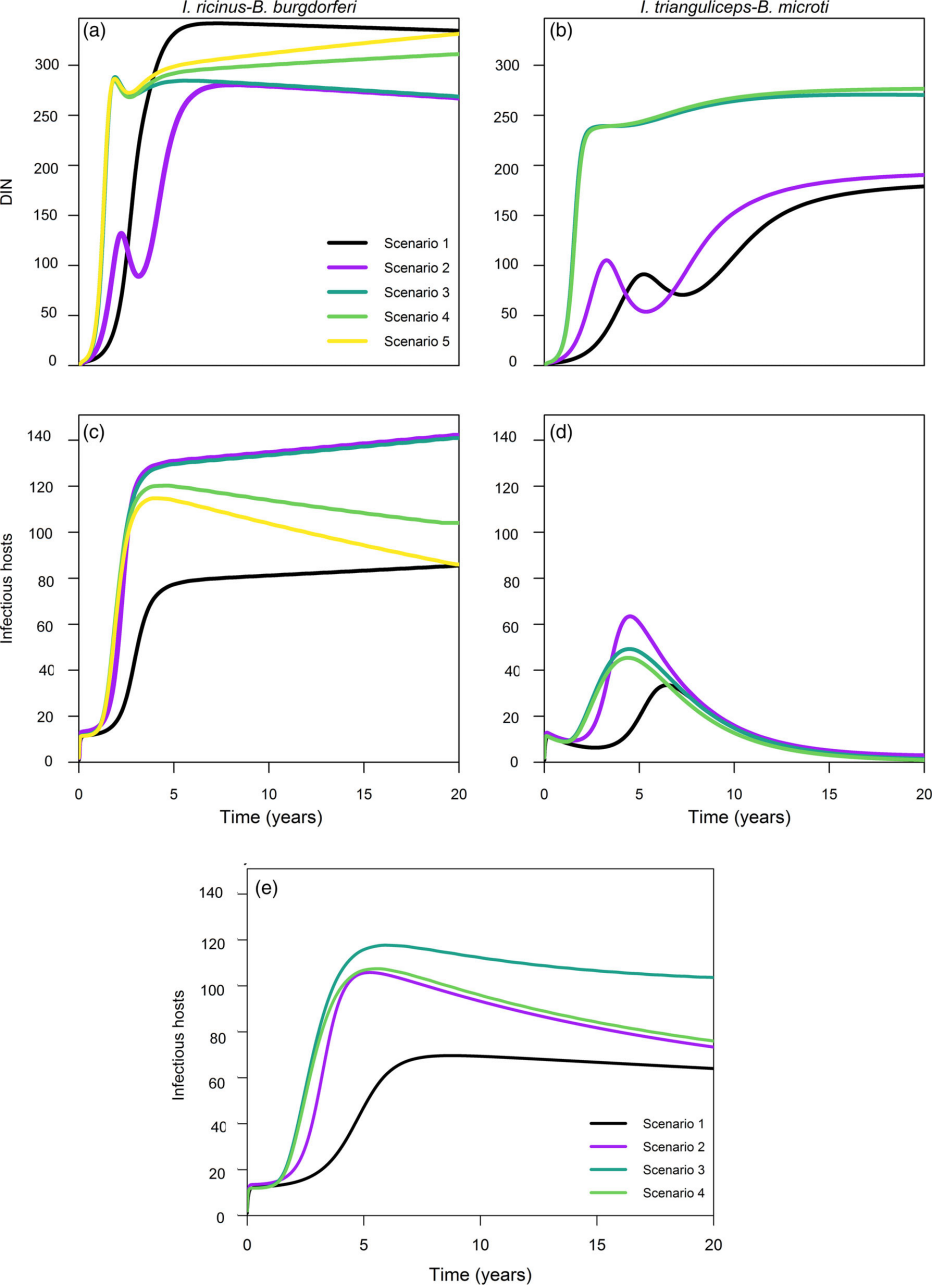
Temporal differences of (a, b) DIN and (c, d) total number of infectious hosts in the two pathosystems over the entire simulation time (20 years). (e) Number of infectious hosts generated over the entire simulation time (20 years) by the specialist tick *I. trianguliceps* transmitting *B. burgdorferi* (modification of the second pathosystem). Black, scenario 1; purple, scenario 2; teal, scenario 3; light green, scenario 4; yellow, scenario 5. Parameter values are listed in Table 2

The percentage of change at equilibria (Figures 2 and 3) suggested that, in both pathosystems, the dilution effect occurred in the competent host populations mostly due to the increase in functional complexity of the community (due to the addition of non‐rodent species from scenario 3 to 5), not by the increase of number of species per se. To appreciate differences over the course of epidemic, Figure 4 shows DIN and number of infectious hosts for each scenario and each pathosystem across the 20 years of simulations (parameters in Table 2).

In the first pathosystem, no dilution could be observed regarding DIN (Figure 4a). Nonetheless, at the start of epidemic, Scenario 1 presents a much higher DIN compared to the other ones, while converging to Scenario 5 at the end. Although the addition of shrews determines a rapid DIN increase compared to Scenario 2, after a few years, despite the overall greater host population, the transmission is slowed down by “wasted bites” at the level of Scenario 2. On the contrary, in the *I. trianguliceps*–*B. microti* system, the addition of more hosts simply increases DIN (although the lower fecundity assumed determines overall smaller tick population and lower DIN; Figure 4b). Looking at the competent host population, no significant dilution occurred in the first pathosystem unless predators were added (Scenario 4), and the addition of ungulates (Scenario 5) further reduced the number of infectious hosts as they were dead ends in terms of transmission (Figure 4c). Nevertheless, comparing Scenario 2 and 3, it could be appreciated how the remarkable increase in total tick host population due to shrews did not correspond in such increase in infectious hosts, suggesting that the transmission reduction mechanism was an important source of infection dilution (Figure 4c). The *I. trianguliceps*–*B. microti* system presented a significantly lower number of infectious hosts over the entire course of epidemic no matter the scenario compared to the first system (Figure 4d). The epidemic is characterized by a lower peak compared to the first pathosystem and at equilibria only few individual rodents became infectious (Figure 4d). This was due to the lower tick fecundity, the higher tick specialization and most of all due to the much shorter infectious period of *B. microti*. In this case, the addition of a second rodent species (Scenario 2) markedly increased the number of infectious hosts compared to Scenario 1, while the addition of competitors (Scenario 3) and predators (Scenario 4) lowered the epidemic peak compared to Scenario 2 and decreased infectious hosts below the on rodent‐only scenarios after several years (Figure 4d).

To investigate the effect of pathogen infectious period on dilution, and disentangle its effect from tick ecology, simulations were performed assuming *I. trianguliceps* transmitting *B. burgdorferi*. The number of infectious hosts are shown in Figure 4e. Comparing these results with those obtained when we simulated *I. ricinus* transmitting the same pathogen (Figure 4c), we could observe a different course of epidemic, more similar to that in Figure 4c, i.e., a much higher number of infectious hosts compared to Figure 4d (but lower than Figure 4c), and overall amplification when increasing host population available to the tick. Only predation (Scenario 4) decreased infectious hosts compared to the previous scenario, but not at lower levels than Scenarios 1 and 2.

**FIGURE 5:**
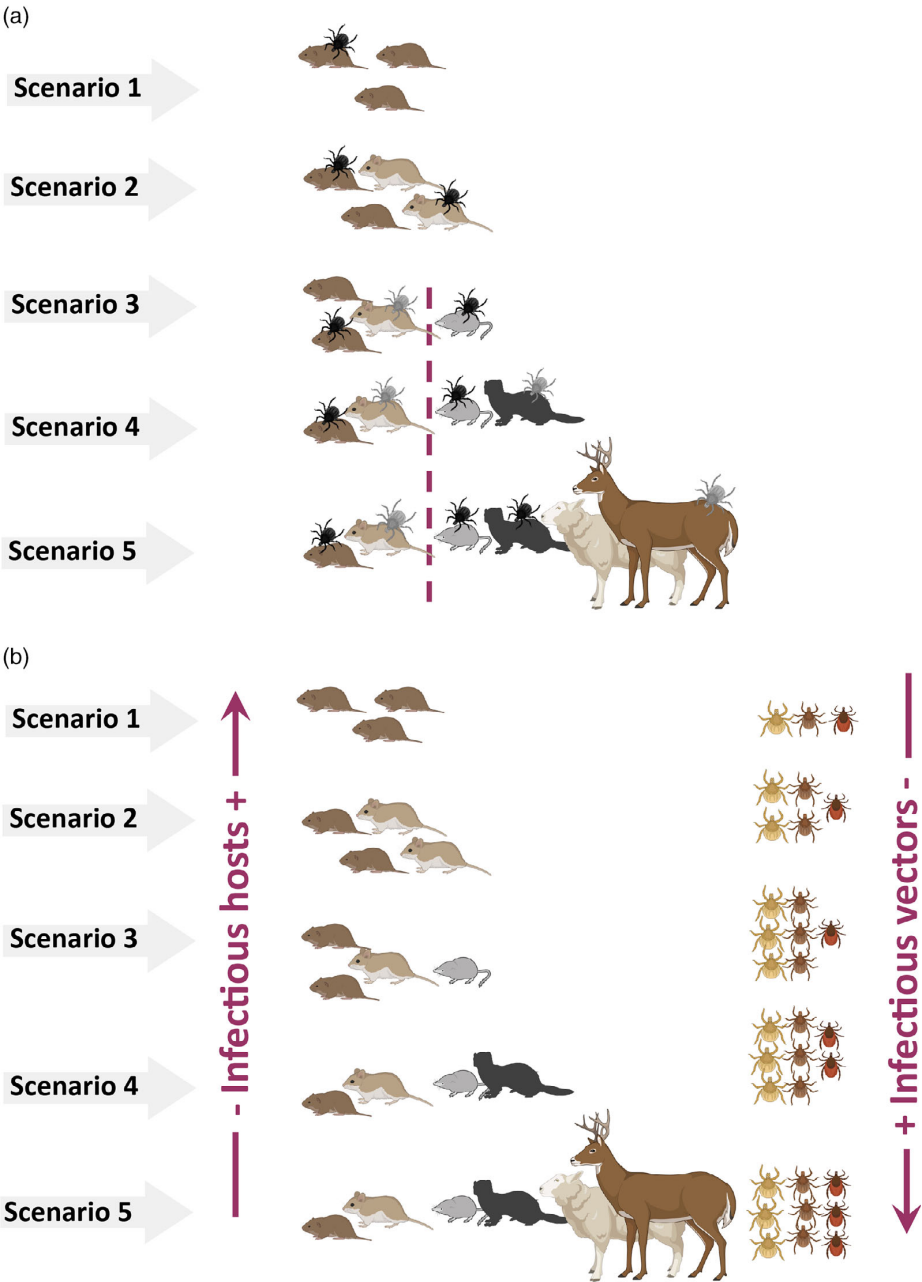
Conceptual model of the dilution mechanisms described by the model. (a) Transmission reduction: addition of non‐competent hosts to the community (scenario 3–5) leads to a reduction in numbers of infectious hosts because of wasted bites. Infectious ticks, in black, feed on non‐competent hosts halting pathogen transmission to hosts (and molting in non‐infectious adults), especially when ticks do not display preferences between competent and non‐competent hosts (e.g., pathosystem with generalist vector). Dashed line separates competent hosts, on the left, from non‐competent hosts, on the right. (b) Susceptible host regulation: increase in community diversity (scenario 1–5) leads to competent host population reduction and so to a reduction of infectious hosts; however, as the number of available hosts for the ticks also rises, there might be a concurrent increase of infectious ticks, especially if ticks feed preferably on competent hosts (e.g., pathosystem with specialist vector)

Thus, it seemed that a sort of hump shaped relationship between community complexity and transmission could be observed for the pathosystem including the generalist vector (*I. ricinus*). Namely, in our simulated scenarios, the single‐host one (Scenario 1) was comparable to the most complex ones (Scenarios 4 and 5), while Scenarios 2 and 3 displayed the peak of the hump (either negative in the case of DIN or positive in the case of infectious hosts). This occurred because ecological relationships (which altered host densities and consequently tick populations) and tick generalism both diluted infections in the most complex communities at the level of the single‐host scenario. This was performed through the mechanisms of transmission reduction (“wasted bites”) and susceptible host regulation (Figure 5). The addition of shrews in Scenario 3 did not impact results as one would expect, considering the significant increase in host population compared to Scenario 2. This is because the high amount of transmission reduction and the density dependent fecundity reduction in ticks did not allow an infinite tick population growth. In Scenarios 4 and 5, amplification and dilution effects occurred simultaneously in vector and host populations respectively.

In the pathosystem with the specialist tick, this relationship was more complex and more dependent upon parameter values. However, amplification occurred when considering DIN, with a progressive increase in infectious nymphs from Scenarios 1 to 4; while dilution could be observed when considering infectious hosts, and was more significant when predation was introduced (Scenario 4). Susceptible host regulation seemed the dilution mechanism more consistent for the pathosystem with the specialist vector. The effect of “wasted bites” observed in the pathosystem with a generalist vector was not crucial here, and determined dilution only when the pathogen infectious period was short.

In summary, there were significant differences between the pathosystems:

1. The host specialization of *I. trianguliceps* (as it fed only on small‐sized hosts) was a significant difference that led to a number of results: (a) a lack of dilution effect with regard to DIN in every scenario; (b) a potential amplification of infectious hosts in Scenario 3 when the shrews, able to sustain tick population, were added to the community.
2. The larger number of “wasted bites” of the generalist *I. ricinus* led to dilution in hosts in each scenario comprising alternative non‐competent hosts (Scenarios 3–5).
3. The generalism of *I. ricinus* (likely combined with the higher fecundity) determined that low molting success or low competence values were sufficient to reach the peak of infections.

## DISCUSSION

### Ground‐dwelling rodent populations survey

The rodent species growth rates estimated were comparable with other live‐trapping studies (Careau et al., 2013; Huitu et al., 2004; Lambin et al., 2000; Merritt et al., 2001). Population densities suggested that field sites hosted rather diverse communities. For most of the sites, a species list including avian and terrestrial predators of our focal species was available, and the mid‐Wales sites were included in a pine marten reintroduction project; shrew feces were recovered in trap tunnels at every site (Occhibove, 2018). Interspecific competition among rodents might also have played a role in keeping population sizes under control, with bank voles seemed to be overall slightly better competitors than wood mice (Occhibove, 2018). This competitive interaction could be hypothesized to be due to the variability in intra and interspecific contacts among rodent species at different sites. This might be explained by different resource and space use, and by the inverse relationship between proportion of interspecific contacts and individual density (Occhibove, 2018).

The numbers of ticks recovered from rodents were in agreement with other studies in which collection was made on living, anesthetized individuals (e.g., Paziewska et al., 2010). The proportion of the population parasitized by ticks and fleas was very small, supporting the “20/80 Rule,” which means ticks were highly clustered on a relatively small proportion of the host population (Occhibove, 2018). In general, bank voles were more parasitized than wood mice, which have been observed to be more heavily parasitized by *I. trianguliceps* than murine species in England (Hussein, 1980). A low number of *I. ricinus* was found at the sampled sites, but this does not exclude the potential presence at nearby sites, as a very fine‐scale distribution of this species in various habitats was observed in the UK (Dobson et al., 2011). Indeed, tick distribution seems to be extremely random and patchy, being determined by local host–vector interaction dynamics (Lorusso et al., 2011); while the ability to recover them it is highly dependent on time and type of sampling, which might hide the real tick species composition (Dobson et al., 2011). Another explanation might be that at our field sites the most frequent ungulate species were sheep and cattle, while deer were absent, and the latter were found more positively associated with *I. ricinus* presence in the UK (Medlock et al., 2008). So, the higher prevalence in bank voles may be determined by the overall dominance of *I. trianguliceps*, since, when *I. ricinus* has been found to be the dominant tick species, wood mice were the most parasitized hosts (e.g., Gray et al., 1999; Kurtenbach et al., 1995).

### Model simulations

This study integrated empirical data about local host communities with mechanistic models to develop a realistic eco‐epidemiological approach for the investigation of tick‐borne pathogens in pathosystems differing in vector degree of generalism. Our main result was the identification of a hump‐shaped relationship between community complexity and transmission in the pathosystem including the generalist vector (*I. ricinus*). This was due to the interaction of ecological relationships, which altered host densities and consequently tick populations, and tick generalism that determined dilution of infection through the mechanisms of transmission reduction (“wasted” bites) and susceptible host regulation (Figure 5). Conversely, in the pathosystem with the specialist tick, this relationship was more complex and seemed to be variable depending on parameter values. In both cases, dilution occurred when considering the host population but not the vectors.

*Ixodes ricinus*, which displays a higher degree of host generalism (Mysterud et al., 2015), seemed to realize a higher number of “wasted” bites, in terms of pathogen transmission, while *I. trianguliceps* largely fed on competent hosts. Nonetheless, a lower value of molting success on rodents (the competent hosts), compared to *I. trianguliceps*, was sufficient to saturate transmission in hosts due to the greater population. On the contrary, the *I. trianguliceps* smaller population determined that a higher value of molting success was necessary to saturate transmission in hosts. In this pathosystem, molting success on rodents appeared to be the predominant regulator of pathogen transmission. Whereas, competence of transmission was crucial in determining the epidemic size only in the scenarios where fewer competent hosts were available. In general, in both pathosystems, variability in competence of transmission vector to rodents resulted in less variability in DIN and infectious hosts compared to molting success hosts, suggesting that competence of focal hosts might not be a limiting factor to transmission.

Our model highlighted the differences in the two host–vector–pathogen systems, justifying the independent modeling of the two pathosystems and supporting the empirical observation of tick and pathogen ecological differences (Mysterud et al., 2015). Our simulations revealed vector population density and proportion between life stages to be comparable with other studies (Dobson et al., 2011; Harrison et al., 2011). The numbers of infectious individuals predicted by our model were plausible in both pathosystems, with *B. burgdorferi* s.l. being significantly more prevalent than *B. microti* (Hussein, 1980; Obiegala et al., 2017; Welc‐Falęciak et al., 2008).

The first dilution mechanism detected in our model was transmission reduction (Figure 5a). This was observed after the introduction of non‐competent hosts into the community, e.g., shrews, which provided a source of “wasted” tick bites (Halsey et al., 2018). This was more marked in the *I. ricinus*–*B. burgdorferi* system, as the generalist tick fed also on the larger hosts included in Scenarios 4 and 5. In this pathosystem, despite the remarkable rise of overall hosts available for ticks, there was no such increase in infectious hosts, although the actual number of infectious nymphs was amplified. In more diverse community assemblages, the greater host availability for the vector led to a rise in the overall vector population, including infectious vectors (amplification effect), but infectious hosts decreased again, suggesting that alternative non‐competent hosts are an effective source of “wasted” bites. This phenomenon could be appreciated because we took into account the actual number of infectious vectors instead of prevalence. Prevalence is more frequently reported in the literature, but generally leads to misleading interpretation of “spurious” dilution results (Dobson & Auld, 2016; Roche et al., 2013). Nevertheless, the dilution potential of a non‐competent host population depends on its relative abundance, degree of tick infestation, interspecific competition and competence; and this requires it to be evaluated in more detail at local scale, as shown in other pathosystems (Mysterud et al., 2019). Most likely this mechanism was detectable due to the assumption of partial species additivity, and the simulation of tick aggregation, which realistically modeled the uneven distribution of ticks across the entire host population (Levi, Keesing, et al., 2016).

In the pathosystem involving the generalist tick, this mechanism acted in synergy with the second dilution mechanism observed, susceptible host regulation (Figure 5b), which in our model was mainly caused by predation (Levi et al., 2012; Ostfeld & Holt, 2004). Yet, dilution due to susceptible host regulation was observed in the specialist tick pathosystem as well. Predators are able to maintain rodent prey at low abundance across a wide range of resource availability (Korpimäki et al., 2004), and have been found to have additionally indirect negative effects on transmission (Hofmeester et al., 2017). In particular, mustelids do not markedly contribute to increases in the tick population (Lorusso et al., 2011; Meyer‐Kayser et al., 2012), suggesting that preserving top‐down forces as predators might be beneficial in decreasing disease risk (Johnson et al., 2019). In different communities, top predators were also observed to regulate deer populations (Korpimäki et al., 2004).

The role of ungulates in transmission dynamics might be more complex than in our model (Kilpatrick et al., 2017), but the effect of large grazing ungulates on ground‐dwelling small mammals’ behavior was beyond our scope, and it is still under investigation (Navarro‐Castilla et al., 2017). Nonetheless, we tested the effect on pathogen transmission of a large‐sized host population, such as sheep or deer, as this is preferred by *I. ricinus* adults and considered to be a source of Lyme disease amplification (Eisen et al., 2012). Our results were in agreement with Huang et al. (2019): i.e., the nymphal infection prevalence reduction outweighed tick population amplification, and this was observed in particular in highly biodiverse contexts (Sprong et al., 2020). Considering the generalist vector system and assuming low density, despite the contribution to a minor DIN increase, the ungulate population diluted transmission in hosts. Further analyses are needed to investigate the relationship between deer abundance and human disease risk at different relative densities (Huang et al., 2019).

A less significant reduction of susceptible hosts (i.e., rodents) resulted from shrew competition, but we chose a conservative estimate, so this remains an important area of uncertainty that requires further investigation (likewise competition among rodent species). In a directly transmitted disease, shrew density has been observed to be negatively correlated to infection probability in the focal rodent host, although the density of the two species were positively related. This suggests that the role of shrews as diluters might include being a source of wasted bites for ticks and a reservoir host competitor, and they may also alter the reservoir host behavior and habitat utilization (Khalil et al., 2016). Information on this aspect might be crucial to better evaluate the role of such competitor species in cases such as our small mammal specialist vector pathosystem. Our results showed that, in the pathosystem characterized by the small mammal specialist tick, the role of shrews as diluter or amplifier was complex and fluctuated as parameters related to the vector or the pathogen varied.

Our results support the findings of Ruyts et al. (2016, 2018), who also identified a sort of hump‐shaped DIN response to the increase in community diversity, together with an overall increase of nymphal population, although they did not test the relationship mechanistically, and assumed that community diversity was correlated to habitat complexity. From their results, it might be expected that the public health risk associated with Lyme disease transmitted by *I. ricinus* in Europe is not decreased by higher diversity, in disagreement with what was found in the United States. This is likely due to the differences between the ecology of tick species involved in those pathosystems (characterized mainly by *I. scapularis*), and the differences in host communities. Nevertheless, in the United States, the relative density of the reservoir host was observed to modulate the effects of species richness on DIN and *B. burgdorferi* host prevalence. So much so that high biodiversity did not always reduce those effects, while the best predictors of tick abundance were deer abundance and temperature at ground level (Werden et al., 2014). This suggests that despite pathosystem and community differences, patterns of community (dis)assembly and the way in which relative densities of competent and non‐competent hosts respond to these patterns might be a common trait to investigate in the context of the diversity–disease relationship.

This relationship is likely to be nonlinear and may be unimodal with a peak at some intermediate level of diversity (Strauss et al., 2016), therefore the European and North American communities might be at different sides of the peak. Very degraded habitats or very low‐diversity communities might have such few host species remaining that the likelihood of some pathogens persisting is extremely low (e.g., Gray et al., 1995; Richter & Matuschka, 2006). Therefore, to reduce disease risk by conserving or restoring biodiversity, it is crucial to determine on which side of the peak a given location falls before attempting an intervention (Strauss et al., 2016). For example, in Britain and Ireland, low‐biodiversity tick habitats are common where grass‐fed livestock are predominant and can alone maintain *I. ricinus* populations; here, low biodiversity can decrease rather than increase the abundance of infected ticks, contrasting with what is observed in the United States (LoGiudice et al., 2003), and emphasizing the complexity of the diversity–human‐disease risk of *B. burgdorferi* s.l. in Europe (Gray et al., 2021). Whereas, in habitats where the overall diversity might be increased by nonnative species, these might represent tick population ecological boosters, potentially increasing disease risk (e.g., Doi et al., 2021).

Considering the differences we have observed modeling the two pathosystems, the variety of aspects of tick ecology might also be crucial to determine disease risk in such pathosystems. For example, *I. ricinus* is more prolific than *I. trianguliceps* (Bown et al., 2006). It determines an overall larger infectious tick population despite the density‐dependent fecundity reduction function modeled and the greater effect of dilution mechanisms. With regard to tick specialization, this has been observed to follow a pattern of global generalism and local specialism, which needs to be better investigated to better understand the circulation of tick‐borne pathogens and exposure risks for humans and livestock (McCoy et al., 2013). Anthropogenic disturbance, and consequent habitat degradation, seems to favor generalist over specialist parasites (just like it has been observed in free living species; Dharmarajan et al., 2021), and this might be the case for vectors and particularly for ticks as well. However, if biotic homogenization of parasite communities usually leads to an increase in disease emergence risk, then pathosystems characterized by generalist ticks could be considered to be the keystone for the dilution effect hypothesis. Another aspect to be taken into account is vector feeding preferences in multi‐host communities and how it aggregates on hosts with different transmission competence levels. It has been demonstrated that this changes with host diversity, decreasing overall pathogen spread due to nonrandom sorting of viruliferous vectors between preferred and non‐preferred host species (Shoemaker et al., 2019).

Our model presented some limitations; first, it did not account for co‐infection, i.e., multiple pathogens influencing relative prevalence (e.g., Diuk‐Wasser et al., 2016; Hersh et al., 2014). Second, it was not spatially explicit, i.e., it did not include links between tick population dynamics and habitat (Maaz et al., 2018), the effect of patch connectivity, individual dispersion, and meta‐populations on pathogen transmission (Cohen et al., 2016), nor the influence of spatial scale and latitude (Magnusson et al., 2020), but these were beyond the scope of the study. Yet, the importance of considering spatial scale in evaluating zoonotic risk has been extensively demonstrated, especially in the case of Lyme disease in the context of forest fragmentation (e.g., Allan et al., 2003; LoGiudice et al., 2008). Third, despite collecting empirical data from wild rodents, crucial epidemiological parameters remained uncertain in our models. Uncertain parameters make unreliable quantitative predictions. Nonetheless, their inclusion in models does highlight important knowledge gaps and this indicates which experimental studies have the most potential to improve modeling predictions (Johnson, de Roode, et al., 2015). However, despite the uncertainty we were still able to identify two distinct dilution mechanisms, the species responsible for each mechanism to occur, and the differences in terms of community‐complexity–transmission relationship in the two pathosystems characterized by generalist and specialist vectors. Focusing on the qualitative patterns of our results, we demonstrated that dilution and amplification effects are not mutually exclusive in the same community, and that they depend on the epidemiological metric under consideration. Our modeling approach was innovative as it expanded from purely additive versus purely substitutive community structures (Johnson, Ostfeld, et al., 2015), creating more ecologically sound communities and a framework that embraced both theoretical and observational aspects (Mihaljevic et al., 2014).

The consistency of our main results with other European studies, i.e., that the density of infected ticks is hardly affected by dilution, supports the idea that in Europe the best strategy to lower tick‐borne disease risk might be the reduction of the tick‐human contact rate. This may be achieved through actions such as guided visitor flows in forests or frequently mowed forest trails instead of trying intervention to generically increase biodiversity (Ruyts et al., 2018). Managing disease risk through dilution might be very difficult for several reasons: when hosts compete for resources reintroduction of diluters might cause undesirable effects on the density of focal hosts; or diluters might fail to control disease in focal hosts because they are constrained by competition (e.g., Becker et al., 2014; Lacroix et al., 2014); or in extreme cases spillover events might occur (Strauss et al., 2015). Additionally, local species interactions, which may potentially interfere with disease transmission, may also exact other ecological consequences. Strauss et al. (2016) found that the observed disease risk–diversity correlation was spurious, and derived from differences in habitat structure, thus they suggest that another option to manage disease risk is to restore habitats. Indeed, in the context of rodent‐borne diseases, if the goal is to minimize disease emergence/abundance without species‐specific knowledge, preserving habitat may be a useful strategy, as land‐use change has been connected to a significant rise in rodent zoonotic reservoir species abundance (Mendoza et al., 2020). This approach might be the most effective option, not only relative to human disease risk, but also in case the pathogen under consideration was to threaten host populations. According to our results, preserving species functional diversity, and in particular top‐down predation, was crucial for the dilution effect to occur in host populations, therefore conserving habitat structure might be effective in avoiding local predator extinctions, and to increase functional diversity in general. Future research might focus on establishing the reservoir role of host/vector species in different habitat types, the behavior of ticks and hosts in different forest types and vegetation features, and the influence of habitat specific properties to identify potential generalities (Kilpatrick et al., 2017).

In conclusion, this investigation into the influence of multiple and diverse ecological components on pathogen transmission identified distinct dilution mechanisms and the species responsible for each mechanism to occur. In both pathosystems simulated, we demonstrated that more complex communities led to fewer infectious hosts providing evidence for dilution. Nonetheless, especially in the system with the more generalist vector, *I. ricinus*, the increase of host availability led to an amplification of human disease risk via the increased density of infectious nymphs (DIN). This was important in the system with the specialist vector only when considering the addition of shrews (potentially alternative hosts); yet, our conservative approach in terms of interspecific competition might have hindered the dilution potential of the competitor population in that system. Hence, our results supported the observations that dilution and amplification effects might not be mutually exclusive in the same community or pathosystem (Halsey, 2019), and that the relationship between diversity and disease risk is nonlinear (Ruyts et al., 2016; Strauss et al., 2016). The interpretation of this relationship depends on the epidemiological metric selected according to the focus of the study (Johnson & Thieltges, 2010; Lou et al., 2014), and patterns of community (dis)assembly in terms of both species identity (i.e., functional role in the community) and relative variation of reservoir species density (Johnson et al., 2019). Instead of trying to find a one‐size‐fits‐all approach to understanding disease–diversity relationships, our results emphasize the value of using eco‐epidemiological modeling supported by empirical data collection to investigate specific pathosystems. In particular, to improve the assessment of wildlife and human disease risk, there is an urgent need for more refined mechanistic approaches and local studies to avoid global ecological fallacy (Kilpatrick et al., 2017). This is crucial to plan ecological interventions to improve wildlife management and zoonotic risk control policies, and to achieve current public health and conservation goals (Sokolow et al., 2019). Currently, without specific knowledge on any given pathosystem the best strategy to achieve these goals seems to be habitat structure conservation.

## CONFLICT OF INTEREST

The authors declare no conflict of interest.

## Supporting information


Appendix S1

Appendix S2

Appendix S3

Data S1

## Data Availability

Code is provided as Supporting Information in Data S1 and is also publicly available in Zenodo at https://doi.org/10.5281/zenodo.5585343 (Occhibove, 2021).

## References

[eap2550-bib-0001] Allan, B. F. , F. Keesing , and R. Ostfeld . 2003. “Effect of Forest Fragmentation on Lyme Disease Risk.” Conservation Biology 17: 267–72.

[eap2550-bib-0002] Anthony, N. , and C. Ribic . 2005. “Comparative Effectiveness of Longworth and Sherman Live Traps.” Wildlife Society Bulletin 33: 1018–26.

[eap2550-bib-0003] Arino, O. , A. El Abdllaoui , J. Mikram , and J. Chattopadhyay . 2004. “Infection in Prey Population May Act as a Biological Control in Ratio‐Dependent Predator‐Prey Models.” Nonlinearity 17: 1101–16.

[eap2550-bib-0004] Becker, G. C. , D. Rodriguez , L. Felipe Toledo , A. V. Longo , C. Lambertini , D. T. Corrêa , D. S. Leite , C. F. B. Haddad , and K. R. Zamudio . 2014. “Partitioning the Net Effect of Host Diversity on an Emerging Amphibian Pathogen.” Proceedings of the Royal Society B 281: 1–7. 10.1098/rspb.2014.1796 PMC4213625 25297867

[eap2550-bib-0005] Begon, M. , S. M. Hazel , D. Baxby , K. Bown , R. Cavanagh , J. Chantrey , T. Jones , and M. Bennett . 1999. “Transmission Dynamics of a Zoonotic Pathogen within and between Wildlife Host Species.” Proceedings of the Royal Society B 266: 1939–45. 10584336 10.1098/rspb.1999.0870 PMC1690313

[eap2550-bib-0006] Bolzoni, L. , G. A. de Leo , M. Gatto , and A. P. Dobson . 2008. “Body‐Size Scaling in an SEI Model of Wildlife Diseases.” Theoretical Population Biology 73: 374–82. 18241903 10.1016/j.tpb.2007.12.003

[eap2550-bib-0007] Bown, K. J. , M. Begon , M. Bennett , R. J. Birtles , S. Burthe , X. Lambin , S. Telfer , Z. Woldehiwet , and N. H. Ogden . 2006. “Sympatric *Ixodes trianguliceps* and *Ixodes ricinus* Ticks Feeding on Field Voles (*Microtus agrestis*): Potential for Increased Risk of *Anaplasma phagocytophilum* in the United Kingdom?” Vector‐Borne and Zoonotic Diseases 6: 404–10. 17187576 10.1089/vbz.2006.6.404

[eap2550-bib-0008] Bown, K. J. , X. Lambin , G. R. Telford , N. H. Ogden , S. Telfer , Z. Woldehiwet , and R. J. Birtles . 2008. “Relative Importance of *Ixodes ricinus* and *Ixodes trianguliceps* as Vectors for *Anaplasma phagocytophilum* and *Babesia microti* in Field Vole (*Microtus agrestis*) Populations.” Applied and Environmental Microbiology 74: 7118–25. 18820068 10.1128/AEM.00625-08 PMC2592922

[eap2550-bib-0009] Brearley, G. , J. Rhodes , A. Bradley , G. Baxter , L. Seabrook , D. Lunney , Y. Liu , and C. McAlpine . 2013. “Wildlife Disease Prevalence in Human‐Modified Landscapes.” Biological Reviews of the Cambridge Philosophical Society 88: 427–42. 23279314 10.1111/brv.12009

[eap2550-bib-0010] Burthe, S. J. , X. Lambin , S. Telfer , A. Douglas , P. Beldomenico , A. Smith , and M. Begon . 2010. “Individual Growth Rates in Natural Field Vole, *Microtus agrestis*, Populations Exhibiting Cyclic Population Dynamics.” Oecologia 162: 653–61. 19916066 10.1007/s00442-009-1495-6

[eap2550-bib-0011] Careau, V. , P. Bergeron , D. Garant , D. Réale , J. R. Speakman , and M. M. Humphries . 2013. “The Energetic and Survival Costs of Growth in Free‐Ranging Chipmunks.” Oecologia 171: 11–23. 22692385 10.1007/s00442-012-2385-x

[eap2550-bib-0012] Civitello, D. J. , and R. B. Hartman . 2021. “Size‐Asymmetric Competition Among Snails Disrupts Production of Human‐Infectious *Schistosoma mansoni* Cercariae.” Ecology 102: e03383. 33950517 10.1002/ecy.3383 PMC8249335

[eap2550-bib-0013] Cohen, J. M. , D. J. Civitello , A. J. Brace , E. M. Feichtinger , C. N. Ortega , J. C. Richardson , E. L. Sauer , X. Liu , and J. R. Rohr . 2016. “Spatial Scale Modulates the Strength of Ecological Processes Driving Disease Distributions.” Proceedings of the National Academy of Sciences USA 113: 3359–64. 10.1073/pnas.1521657113 PMC4914148 27247398

[eap2550-bib-0014] de Leo, G. A. , and P. A. Dobson . 1996. “Allometry and Simple Epidemic Models for Microparasites.” Nature 379: 720–2. 8602216 10.1038/379720a0

[eap2550-bib-0015] Dharmarajan, G. , P. Gupta , C. K. Vishnudas , and V. V. Robin . 2021. “Anthropogenic Disturbance Favours Generalist over Specialist Parasites in Bird Communities: Implications for Risk of Disease Emergence.” Ecology Letters 24: 1859–68. 34120404 10.1111/ele.13818

[eap2550-bib-0016] Diuk‐Wasser, M. A. , E. Vannier , and P. J. Krause . 2016. “Coinfection by *Ixodes* Tick‐Borne Pathogens: Ecological, Epidemiological, and Clinical Consequences.” Trends in Parasitology 32: 30–42. 26613664 10.1016/j.pt.2015.09.008 PMC4713283

[eap2550-bib-0017] Dobson, A. 2004. “Population Dynamics of Pathogens with Multiple Host Species.” American Naturalist 164((Suppl 5)): S64–78. 10.1086/424681 15540143

[eap2550-bib-0018] Dobson, A. D. M. , and S. K. J. R. Auld . 2016. “Epidemiological Implications of Host Biodiversity and Vector Biology: Key Insights from Simple Models.” American Naturalist 187: 405–22. 10.1086/685445 27028070

[eap2550-bib-0019] Dobson, A. D. M. , J. L. Taylor , and S. E. Randolph . 2011. “Tick (*Ixodes ricinus*) Abundance and Seasonality at Recreational Sites in the UK: Hazards in Relation to Fine‐Scale Habitat Types Revealed by Complementary Sampling Methods.” Ticks and Tick‐Borne Diseases 2: 67–74. 21771540 10.1016/j.ttbdis.2011.03.002

[eap2550-bib-0020] Doi, K. , M. Kono , T. Kato , and S. Hayama . 2021. “Ecological Traps and Boosters of Ixodid Ticks: The Differing Ecological Roles of Two Sympatric Introduced Mammals.” Ticks and Tick‐Borne Diseases 12: 101687. 33631488 10.1016/j.ttbdis.2021.101687

[eap2550-bib-0021] Dunn, J. M. , S. Davis , A. Stacey , and M. A. Diuk‐Wasser . 2013. “A Simple Model for the Establishment of Tick‐Borne Pathogens of Ixodes Scapularis: A Global Sensitivity Analysis of R_0_ .” Journal of Theoretical Biology 21: 213–21. 10.1016/j.jtbi.2013.06.035 PMC3913058 23850477

[eap2550-bib-0022] Dunn, R. R. , T. J. Davies , N. C. Harris , and M. C. Gavin . 2010. “Global Drivers of Human Pathogen Richness and Prevalence.” Proceedings of the Royal Society B 277: 2587–95. 20392728 10.1098/rspb.2010.0340 PMC2982038

[eap2550-bib-0023] Eisen, R. J. , J. Piesman , E. Zielinski‐Gutierrez , and L. Eisen . 2012. “What Do we Need to Know about Disease Ecology to Prevent Lyme Disease in the Northeastern United States?” Journal of Medical Entomology 49: 11–22. 22308766 10.1603/me11138

[eap2550-bib-0024] Erlinge, S. 1975. “Feeding Habits of the Weasel *Mustela nivalis* in Relation to Prey Abundance.” Oikos 26: 378–84.

[eap2550-bib-0025] Faust, C. L. , A. P. Dobson , N. Gottdenker , L. S. P. Bloomfield , H. I. McCallum , T. R. Gillespie , M. A. Diuk‐Wasser , and R. K. Plowright . 2017. “Null Expectations for Disease Dynamics in Shrinking Habitat: Dilution or Amplification?” Philosophical Transactions of the Royal Society B 372: 20160173. 10.1098/rstb.2016.0173 PMC5413880 28438921

[eap2550-bib-0026] Ferreri, L. , M. Giacobini , P. Bajardi , L. Bertolotti , L. Bolzoni , V. Tagliapietra , A. Rizzoli , and R. Rosà . 2014. “Pattern of Tick Aggregation on Mice: Larger than Expected Distribution Tail Enhances the Spread of Tick‐Borne Pathogens.” PLoS Computational Biology 10: e1003931. 25393293 10.1371/journal.pcbi.1003931 PMC4230730

[eap2550-bib-0027] Giardina, A. R. , K. A. Schmidt , E. M. Schauber , and R. S. Ostfeld . 2000. “Modeling the Role of Songbirds and Rodents in the Ecology of Lyme Disease.” Canadian Journal of Zoology 78: 2184–97.

[eap2550-bib-0028] Gray, J. , O. Kahl , and A. Zintl . 2021. “What Do we Still Need to Know about Ixodes Ricinus?” Ticks and Tick‐Borne Diseases 12: 101682. 33571753 10.1016/j.ttbdis.2021.101682

[eap2550-bib-0029] Gray, J. S. 2006. “Identity of the Causal Agents of Human Babesiosis in Europe.” International Journal of Medical Microbiology 296: 131–6. 16524772 10.1016/j.ijmm.2006.01.029

[eap2550-bib-0030] Gray, J. S. , O. Kahl , C. Janetzki , J. Stein , and E. Guy . 1995. “The Spatial Distribution of Borrelia Burgdorferi‐Infected Ixodes Ricinus in the Connemara Region of County Galway, Ireland.” Experimental and Applied Acarology 19: 163–72. 7634971 10.1007/BF00046288

[eap2550-bib-0031] Gray, J. S. , F. Kirstein , J. N. Robertson , J. Stein , and O. Kahl . 1999. “Borrelia Burgdorferi Sensu Lato in Ixodes Ricinus Ticks and Rodents in a Recreational Park in South‐Western Ireland.” Experimental and Applied Acarology 23: 717–29. 10581711 10.1023/a:1006233700194

[eap2550-bib-0032] Halsey, S. 2019. “Defuse the Dilution Effect Debate.” Nature Ecology and Evolution 3: 145–6. 30532044 10.1038/s41559-018-0764-3

[eap2550-bib-0033] Halsey, S. J. , B. F. Allan , and J. R. Miller . 2018. “The Role of *Ixodes scapularis*, *Borrelia burgdorferi* and Wildlife Hosts in Lyme Disease Prevalence: A Quantitative Review.” Ticks and Tick‐Borne Diseases 9: 1103–14. 29680260 10.1016/j.ttbdis.2018.04.006

[eap2550-bib-0034] Han, B. A. , J. P. Schmidt , S. E. Bowden , and J. M. Drake . 2015. “Rodent Reservoirs of Future Zoonotic Diseases.” Proceedings of the National Academy of Sciences USA 112: 7039–44. 10.1073/pnas.1501598112 PMC4460448 26038558

[eap2550-bib-0035] Hancock, P. A. , R. Brackley , and S. C. F. Palmer . 2011. “Modelling the Effect of Temperature Variation on the Seasonal Dynamics of Ixodes Ricinus Tick Populations.” International Journal for Parasitology 41: 513–22. 21295037 10.1016/j.ijpara.2010.12.012

[eap2550-bib-0036] Hanski, I. , and H. Henttonen . 1996. “Predation on Competing Rodent Species: A Simple Explanation of Complex Patterns.” Journal of Animal Ecology 65: 220–32.

[eap2550-bib-0037] Hanski, I. , P. Turchin , E. Korpimäki , and H. Henttonen . 1993. “Population Oscillations of Boreal Rodents: Regulation by Mustelid Predators Leads to Chaos.” Nature 364: 232–5. 8321318 10.1038/364232a0

[eap2550-bib-0038] Harrison, A. , W. I. Montgomery , and K. J. Bown . 2011. “Investigating the Persistence of Tick‐Borne Pathogens Via the R₀ Model.” Parasitology 138: 896–905. 21518464 10.1017/S0031182011000400

[eap2550-bib-0039] Hartemink, N. A. , S. E. Randolph , S. A. Davis , and J. A. P. Heesterbeek . 2008. “The Basic Reproduction Number for Complex Disease Systems: Defining R_0_ for Tick‐Borne Infections.” American Naturalist 171: 743–54. 10.1086/587530 18462128

[eap2550-bib-0040] Henttonen, H. , V. Haukisalmi , A. Kaikusalo , E. Korpimäki , K. Norrdahl , and U. A. P. Skarén . 1989. “Long‐Term Population Dynamics of the Common Shrew *Sorex araneus* in Finland.” Annales Zoologici Fennici 26: 349–55.

[eap2550-bib-0041] Hersh, M. H. , R. S. Ostfeld , D. J. McHenry , M. Tibbetts , J. L. Brunner , M. E. Killilea , K. LoGiudice , K. A. Schmidt , and F. Keesing . 2014. “Co‐Infection of Blacklegged Ticks with *Babesia microti* and *Borrelia burgdorferi* Is Higher than Expected and Acquired from Small Mammal Hosts.” PLoS One 9: e99348. 24940999 10.1371/journal.pone.0099348 PMC4062422

[eap2550-bib-0042] Hofmeester, T. R. , P. A. Jansen , H. J. Wijnen , E. C. Coipan , M. Fonville , H. H. T. Prins , H. Sprong , and S. E. van Wieren . 2017. “Cascading Effects of Predator Activity on Tick‐Borne Disease Risk.” Proceedings of the Royal Society B 284: 20170453. 28724731 10.1098/rspb.2017.0453 PMC5543215

[eap2550-bib-0043] Hörnfeldt, B. 1994. “Delayed Density Dependence as a Determinant of Vole Cycles.” Ecology 75: 791–806.

[eap2550-bib-0044] Huang, C. I. , S. C. Kay , S. Davis , D. M. Tufts , K. Gaffett , B. Tefft , and M. A. Diuk‐Wasser . 2019. “High Burdens of *Ixodes scapularis* Larval Ticks on White‐Tailed Deer May Limit Lyme Disease Risk in a Low Biodiversity Setting.” Ticks and Tick‐Borne Diseases 10: 258–68. 30446377 10.1016/j.ttbdis.2018.10.013 PMC6377166

[eap2550-bib-0045] Huitu, O. , K. Norrdahl , and E. Korpimäki . 2004. “Competition, Predation and Interspecific Synchrony in Cyclic Small Mammal Communities.” Ecography 27: 197–206.

[eap2550-bib-0046] Hussein, H. S. 1980. “ *Ixodes trianguliceps*: Seasonal Abundance and Role in the Epidemiology of *Babesia microti* Infection in North‐Western England.” Annals of Tropical Medicine and Parasitology 74: 531–9. 7008722 10.1080/00034983.1980.11687381

[eap2550-bib-0047] Iman, R. L. , J. C. Helton , and J. E. Campbell . 1981a. “An Approach to Sensitivity Analysis of Computer Models: Part I—Introduction, Input Variable Selection and Preliminary Variable Assessment.” Journal of Quality Technology 13: 174–83.

[eap2550-bib-0048] Iman, R. L. , J. C. Helton , and J. E. Campbell . 1981b. “An Approach to Sensitivity Analysis of Computer Models: Part II—Ranking of Input Variables, Response Surface Validation, Distribution Effect and Technique Synopsis.” Journal of Quality Technology 13: 232–40.

[eap2550-bib-0049] Jacquet, M. , J. Durand , O. Rais , and M. J. Voordouw . 2016. “Strain‐Specific Antibodies Reduce Co‐Feeding Transmission of the Lyme Disease Pathogen, *Borrelia afzelii* .” Environmental Microbiology 18: 833–45. 26411486 10.1111/1462-2920.13065

[eap2550-bib-0050] Johnson, P. T. J. , D. M. Calhoun , T. Riepe , T. McDevitt‐Galles , and J. Koprivnikar . 2019. “Community Disassembly and Disease: Realistic—but Not Randomized—Biodiversity Losses Enhance Parasite Transmission.” Proceedings of the Royal Society B 286: 20190260. 31039724 10.1098/rspb.2019.0260 PMC6532514

[eap2550-bib-0051] Johnson, P. T. J. , J. C. De Roode , and A. Fenton . 2015. “Why Infectious Disease Research Needs Community Ecology.” Science 349: 1259504. 26339035 10.1126/science.1259504 PMC4863701

[eap2550-bib-0052] Johnson, P. T. J. , R. S. Ostfeld , and F. Keesing . 2015. “Frontiers in Research on Biodiversity and Disease.” Ecology Letters 18: 1119–33. 26261049 10.1111/ele.12479 PMC4860816

[eap2550-bib-0053] Johnson, P. T. J. , D. L. Preston , J. T. Hoverman , J. S. Henderson , S. H. Paull , K. L. D. Richgels , and M. D. Redmond . 2012. “Species Diversity Reduces Parasite Infection through Cross‐ Generational Effects on Host Abundance.” Ecology 93: 56–64. 22486087 10.1890/11-0636.1

[eap2550-bib-0054] Johnson, P. T. J. , D. L. Preston , J. T. Hoverman , and B. E. Lafonte . 2013. “Host and Parasite Diversity Jointly Control Disease Risk in Complex Communities.” Proceedings of the National Academy of Sciences USA 110: 16916–21. 10.1073/pnas.1310557110 PMC3800997 24082092

[eap2550-bib-0055] Johnson, P. T. J. , and D. W. Thieltges . 2010. “Diversity, Decoys and the Dilution Effect: How Ecological Communities Affect Disease Risk.” Journal of Experimental Biology 213: 961–70. 20190121 10.1242/jeb.037721

[eap2550-bib-0056] Keesing, F. , R. D. Holt , and R. S. Ostfeld . 2006. “Effects of Species Diversity on Disease Risk.” Ecology Letters 9: 485–98. 16623733 10.1111/j.1461-0248.2006.00885.x

[eap2550-bib-0057] Khalil, H. , F. Ecke , M. Evander , M. Magnusson , and B. Hörnfeldt . 2016. “Declining Ecosystem Health and the Dilution Effect.” Scientific Reports 6: 1–11. 27499001 10.1038/srep31314 PMC4976314

[eap2550-bib-0058] Kilpatrick, A. M. , A. D. M. Dobson , T. Levi , D. J. Salkeld , A. Swei , H. S. Ginsberg , A. Kjemtrup , et al. 2017. “Lyme Disease Ecology in a Changing World: Consensus, Uncertainty and Critical Gaps for Improving Control.” Philosophical Transactions of the Royal Society B 372: 20160117. 10.1098/rstb.2016.0117 PMC5413869 28438910

[eap2550-bib-0059] Korpimäki, E. 1992. “Diet Composition, Prey Choice, and Breeding Success of Long‐Eared Owls: Effects of Multiannual Fluctuations in Food Abundance.” Canadian Journal of Zoology 70: 2373–81.

[eap2550-bib-0060] Korpimäki, E. , P. R. Brown , J. Jacob , and R. P. Pech . 2004. “The Puzzles of Population Cycles and Outbreaks of Small Mammals Solved?” BioScience 54: 1071–9.

[eap2550-bib-0061] Korpimäki, E. , and K. Norrdahl . 1989. “Predation of Tengmalm's Owls: Numerical Responses, Functional Responses and Dampening Impact on Population Fluctuations of Microtines.” Oikos 54: 154–64.

[eap2550-bib-0062] Kovalevskii, Y. V. , E. I. Korenberg , N. B. Gorelova , and V. Nefedova . 2013. “The Ecology of *Ixodes trianguliceps* Ticks and their Role in the Natural Foci of Ixodid Tick‐Borne Borrelioses in the Middle Urals.” Entomological Review 93: 1073–83. 10.1016/j.ttbdis.2015.02.004 25843812

[eap2550-bib-0063] Krasnov, B. R. , M. Stanko , and S. Morand . 2007. “Host Community Structure and Infestation by Ixodid Ticks: Repeatability, Dilution Effect and Ecological Specialization.” Oecologia 154: 185–94. 17684769 10.1007/s00442-007-0824-x

[eap2550-bib-0064] Krebs, C. J. , and J. H. Myers . 1974. “Population Cycles in Small Mammals.” Advances in Ecological Research 8: 267–399.

[eap2550-bib-0065] Kurtenbach, K. , H. Kampen , A. Dizij , S. Arndt , H. M. Seitz , U. E. Schaible , and M. M. Simon . 1995. “Infestation of Rodents with Larval *Ixodes ricinus* (Acari: Ixodidae) Is an Important Factor in the Transmission Cycle of *Borrelia burgdorferi* s.l. in German Woodlands.” Journal of Medical Entomology 32: 807–17. 8551503 10.1093/jmedent/32.6.807

[eap2550-bib-0066] Lacroix, C. , A. Jolles , E. W. Seabloom , A. G. Power , C. E. Mitchell , T. Borer , and E. T. Borer . 2014. “Non‐random Biodiversity Loss Underlies Predictable Increases in Viral Disease Prevalence Non‐random Biodiversity Loss Underlies Predictable Increases in Viral Disease Prevalence.” Journal of the Royal Society Interface 11: 20130947. 24352672 10.1098/rsif.2013.0947 PMC3899862

[eap2550-bib-0067] Lambin, X. , S. J. Petty , and J. L. Mackinnon . 2000. “Cyclic Dynamics in Field Vole Populations and Generalist Predation.” Journal of Animal Ecology 69: 106–19.

[eap2550-bib-0068] Levi, T. , F. Keesing , R. D. Holt , M. Barfield , and R. S. Ostfeld . 2016. “Quantifying Dilution and Amplification in a Community of Hosts for Tick‐Borne Pathogens.” Ecological Applications 26: 484–98. 27209790 10.1890/15-0122

[eap2550-bib-0069] Levi, T. , A. M. Kilpatrick , M. Mangel , and C. C. Wilmers . 2012. “Deer, Predators, and the Emergence of Lyme Disease.” Proceedings of the National Academy of Sciences USA 109: 10942–7. 10.1073/pnas.1204536109 PMC3390851 22711825

[eap2550-bib-0071] Loaiza, J. R. , J. R. Rovira , O. I. Sanjur , J. A. Zepeda , J. E. Pecor , D. H. Foley , L. Dutari , et al. 2019. “Forest Disturbance and Vector Transmitted Diseases in the Lowland Tropical Rainforest of Central Panama.” Tropical Medicine and International Health 24: 849–61. 31095798 10.1111/tmi.13244

[eap2550-bib-0072] LoGiudice, K. , S. T. K. Duerr , M. J. Newhouse , K. A. Schmidt , M. E. Killilea , and R. S. Ostfeld . 2008. “Impact of Host Community Composition on Lyme Disease Risk.” Ecology 89: 2841–9. 18959321 10.1890/07-1047.1

[eap2550-bib-0073] LoGiudice, K. , R. S. Ostfeld , K. A. Schmidt , and F. Keesing . 2003. “The Ecology of Infectious Disease: Effects of Host Diversity and Community Composition on Lyme Disease Risk.” Proceedings of the National Academy of Sciences USA 100: 567–71. 10.1073/pnas.0233733100 PMC141036 12525705

[eap2550-bib-0074] Lorusso, V. , R. P. Lia , F. Dantas‐Torres , E. Mallia , S. Ravagnan , G. Capelli , and D. Otranto . 2011. “Ixodid Ticks of Road‐Killed Wildlife Species in Southern Italy: New Tick‐Host Associations and Locality Records.” Experimental and Applied Acarology 55: 293–300. 21728058 10.1007/s10493-011-9470-4

[eap2550-bib-0075] Lotka, A. J. 1925. Elements of Physical Biology. Philadelphia, PA: Williams and Wilkins Company.

[eap2550-bib-0076] Lou, Y. , J. Wu , and X. Wu . 2014. “Impact of Biodiversity and Seasonality on Lyme‐Pathogen Transmission.” Theoretical Biology and Medical Modelling 11: 50. 25432469 10.1186/1742-4682-11-50 PMC4396072

[eap2550-bib-0077] Maaz, D. , J. Krücken , J. Blümke , D. Richter , J. McKay‐Demeler , F.‐R. Matuschka , S. Hartmann , and G. von Samson‐Himmelstjerna . 2018. “Factors Associated with Diversity, Quantity and Zoonotic Potential of Ectoparasites on Urban Mice and Voles.” PLoS One 13(6): e0199385. 29940047 10.1371/journal.pone.0199385 PMC6016914

[eap2550-bib-0078] Magnusson, M. , I. R. Fischhoff , F. Ecke , B. Hörnfeldt , and R. S. Ostfeld . 2020. “Effect of Spatial Scale and Latitude on Diversity–Disease Relationships.” Ecology 101: e02955. 31840238 10.1002/ecy.2955 PMC7078972

[eap2550-bib-0079] Marino, S. , I. B. Hogue , C. J. Ray , and D. E. Kirschner . 2008. “A Methodology for Performing Global Uncertainty and Sensitivity Analysis in Systems Biology.” Journal of Theoretical Biology 254: 178–96. 18572196 10.1016/j.jtbi.2008.04.011 PMC2570191

[eap2550-bib-0080] McCoy, K. D. , E. Léger , and M. Dietrich . 2013. “Host Specialization in Ticks and Transmission of Tick‐Borne Diseases: A Review.” Frontiers in Cellular and Infection Microbiology 4: 1–12. 10.3389/fcimb.2013.00057 PMC3790072 24109592

[eap2550-bib-0081] Medlock, J. M. , M. E. Pietzsch , N. V. P. Rice , L. Jones , E. Kerrod , D. Avenell , S. Los , N. Ratcliffe , S. Leach , and T. Butt . 2008. “Investigation of Ecological and Environmental Determinants for the Presence of Questing *Ixodes ricinus* (Acari: Ixodidae) on Gower, South Wales.” Journal of Medical Entomology 45: 314–25. 18402148 10.1603/0022-2585(2008)45[314:ioeaed]2.0.co;2

[eap2550-bib-0082] Meerburg, B. G. , G. R. Singleton , and A. Kijlstra . 2009. “Rodent‐Borne Diseases and their Risks for Public Health.” Critical Reviews in Microbiology 35: 221–70. 19548807 10.1080/10408410902989837

[eap2550-bib-0083] Mendoza, H. , A. V. Rubio , G. E. García‐Peña , G. Suzán , and J. A. Simonetti . 2020. “Does Land‐Use Change Increase the Abundance of Zoonotic Reservoirs? Rodents Say Yes.” European Journal of Wildlife Research 66: 6.

[eap2550-bib-0084] Merritt, J. F. , M. Lima , and F. Bozinovic . 2001. “Seasonal Regulation in Fluctuating Small Mammal Populations: Feedback Structure and Climate.” Oikos 94: 505–14.

[eap2550-bib-0085] Meyer‐Kayser, E. , L. Hoffmann , C. Silaghi , K. Pfister , M. Mahling , and L. M. F. Passos . 2012. “Dynamics of Tick Infestations in Foxes in Thuringia, Germany.” Ticks and Tick‐Borne Diseases 3: 232–9. 22884924 10.1016/j.ttbdis.2012.05.004

[eap2550-bib-0086] Mihaljevic, J. R. , M. B. Joseph , S. A. Orlofske , and S. H. Paull . 2014. “The Scaling of Host Density with Richness Affects the Direction, Shape, and Detectability of Diversity‐Disease Relationships.” PLoS One 9: e97812. 24849581 10.1371/journal.pone.0097812 PMC4029764

[eap2550-bib-0087] Mysterud, A. , R. Byrkjeland , L. Qviller , and H. Viljugrein . 2015. “The Generalist Tick Ixodes Ricinus and the Specialist Tick Ixodes Trianguliceps on Shrews and Rodents in a Northern Forest Ecosystem – A Role of Body Size Even Among Small Hosts.” Parasites & Vectors 8. 10.1186/s13071-015-1258-7 PMC4681159 26671686

[eap2550-bib-0088] Mysterud, A. , V. M. Stigum , H. Linløkken , A. Herland , and H. Viljugrein . 2019. “How General Are Generalist Parasites? The Small Mammal Part of the Lyme Disease Transmission Cycle in Two Ecosystems in Northern Europe.” Oecologia 190: 115–26. 31062166 10.1007/s00442-019-04411-2

[eap2550-bib-0089] Navarro‐Castilla, A. , M. Diaz , and I. Barja . 2017. “Does Ungulate Disturbance Mediate Behavioral and Physiological Stress Responses in Algerian Mice (*Mus spretus*)? A Wild Exclosure Experiment.” Hystrix 28: 165–72.

[eap2550-bib-0090] Norman, R. , R. G. Bwers , M. Begon , and P. J. Hudson . 1999. “Persistence of Tick‐Borne Virus in the Presence of Multiple Host Species: Tick Reservoirs and Parasite Mediated Competition.” Journal of Theoretical Biology 200: 111–8. 10479543 10.1006/jtbi.1999.0982

[eap2550-bib-0091] Obiegala, A. , N. Król , C. Oltersdorf , J. Nader , and M. Pfeffer . 2017. “The Enzootic Life‐Cycle of *Borrelia burgdorferi* (Sensu Lato) and Tick‐Borne Rickettsiae: An Epidemiological Study on Wild‐Living Small Mammals and their Ticks from Saxony, Germany.” Parasites and Vectors 10: 1–9. 28285593 10.1186/s13071-017-2053-4 PMC5346851

[eap2550-bib-0092] Occhibove, F. 2018. Observational and Model Evidence for and against the Dilution Effect: Examples from Pathogens and Parasites of Wild Rodents. PhD thesis. Aberystwyth University, Aberystwyth.

[eap2550-bib-0093] Occhibove, F. 2021. Flafflyfreak/Tick_model_OcchiboveEtAl: Tick_borne_model.R (v1.0.0). Zenodo. 10.5281/zenodo.5585344

[eap2550-bib-0094] Ogden, N. H. , M. Bigras‐Poulin , C. J. O'callaghan , I. K. Barker , K. Kurtenbach , L. R. Lindsay , and D. F. Charron . 2007. “Vector Seasonality, Host Infection Dynamics and Fitness of Pathogens Transmitted by the Tick *Ixodes scapularis* .” Parasitology 134: 209–27. 17032476 10.1017/S0031182006001417

[eap2550-bib-0095] O'Regan, S. M. , J. E. Vinson , and A. W. Park . 2015. “Interspecific Contact and Competition May Affect the Strength and Direction of Disease‐Diversity Relationships for Directly Transmitted Microparasites.” American Naturalist 186: 480–94. 10.1086/682721 26655572

[eap2550-bib-0096] Ostfeld, R. S. , and R. D. Holt . 2004. “Are Predators Good for Your Health? Evaluating Evidence for Top‐Down Regulations of Zoonotic Disease Reservoirs.” Frontiers in Ecology and the Environment 2: 13–20.

[eap2550-bib-0097] Ostfeld, R. S. , and F. Keesing . 2000. “Biodiversity and Disease Risk: The Case of Lyme Disease.” Conservation Biology 14: 722–8.

[eap2550-bib-0098] Ostfeld, R. S. , and F. Keesing . 2012. “Effects of Host Diversity on Infectious Disease.” Annual Review of Ecology, Evolution, and Systematics 43: 157–82.

[eap2550-bib-0099] Ostfeld, R. S. , and K. LoGiudice . 2003. “Community Disassembly, Biodiversity Loss, and the Erosion of an Ecosystem Service.” Ecology 84: 1421–7.

[eap2550-bib-0100] Paziewska, A. , P. D. Harris , L. Zwolińska , A. Bajer , and E. Siński . 2012. “Differences in the Ecology of *Bartonella* Infections of *Apodemus flavicollis* and *Myodes glareolus* in a Boreal Forest.” Parasitology 139: 881–93. 22336264 10.1017/S0031182012000170

[eap2550-bib-0101] Paziewska, A. , L. Zwolińska , P. D. Harris , A. Bajer , and E. Siński . 2010. “Utilisation of Rodent Species by Larvae and Nymphs of Hard Ticks (Ixodidae) in Two Habitats in NE Poland.” Experimental and Applied Acarology 50: 79–91. 19421876 10.1007/s10493-009-9269-8

[eap2550-bib-0102] Piesman, J. 1989. “Transmission of Lyme Disease Spirochetes (*Borrelia burgdorferi*).” Experimental and Applied Acarology 7: 71–80. 2667921 10.1007/BF01200454

[eap2550-bib-0103] Pongsiri, M. J. , J. Roman , V. O. Ezenwa , T. L. Goldberg , H. S. Koren , S. C. Newbold , R. S. Ostfeld , S. K. Pattanayak , and D. J. Salkeld . 2009. “Biodiversity Loss Affects Global Disease Ecology.” BioScience 59: 945–54.

[eap2550-bib-0104] Porco, T. C. 1999. “A Mathematical Model of the Ecology of Lyme Disease.” Mathematical Medicine and Biology: A Journal of the IMA 16: 261–96. 10520492

[eap2550-bib-0105] Previtali, M. A. , R. S. Ostfeld , F. Keesing , A. E. Jolles , R. Hanselmann , and L. B. Martin . 2012. “Relationship between Pace of Life and Immune Responses in Wild Rodents.” Oikos 121: 1483–92.

[eap2550-bib-0106] R Core Team . 2016. R: A Language and Environment for Statistical Computing. R‐3.2.5 for Windows (32/64 Bit). Vienna: R Foundation for Statistical Computing. https://www.R-project.org/

[eap2550-bib-0107] Randolph, S. E. 1995. “Quantifying Parameters in the Transmission of *Babesia microti* by the Tick *Ixodes trianguliceps* Amongst Voles (*Clethrionomys glareolus*).” Parasitology 110: 287–95. 7724236 10.1017/s0031182000080872

[eap2550-bib-0108] Randolph, S. E. 2004. “Tick Ecology: Processes and Patterns Behind the Epidemiological Risk Posed by Ixodid Ticks as Vectors.” Parasitology 129: S37–65. 15938504 10.1017/s0031182004004925

[eap2550-bib-0109] Randolph, S. E. , and A. D. M. Dobson . 2012. “Pangloss Revisited: A Critique of the Dilution Effect and the Biodiversity‐Buffers‐Disease Paradigm.” Parasitology 139: 847–63. 22336330 10.1017/S0031182012000200

[eap2550-bib-0110] Randolph, S. E. , L. Gern , and P. A. Nuttall . 1996. “Co‐Feeding Ticks: Epidemiological Significance for Tick‐Borne Pathogen Transmission.” Parasitology Today 12: 472–9. 15275266 10.1016/s0169-4758(96)10072-7

[eap2550-bib-0111] Richter, D. , and F. R. Matuschka . 2006. “Modulatory Effect of Cattle on Risk for Lyme Disease.” Emerging Infectious Diseases 12: 1919–23. 17326945 10.3201/eid1212.051552 PMC3291337

[eap2550-bib-0112] Roche, B. , P. Rohani , A. P. Dobson , and J.‐F. Guégan . 2013. “The Impact of Community Organization on Vector‐Borne Pathogens.” American Naturalist 181: 1–11. 10.1086/668591 23234841

[eap2550-bib-0113] Rogalski, M. A. , C. D. Gowler , C. L. Shaw , R. A. Hufbauer , and M. A. Duffy . 2017. “Human Drivers of Ecological and Evolutionary Dynamics in Emerging and Disappearing Infectious Disease Systems.” Philosophical Transactions of the Royal Society B 372: 20160043. 10.1098/rstb.2016.0043 PMC5182439 27920388

[eap2550-bib-0114] Rohr, J. R. , D. J. Civitello , P. W. Crumrine , N. T. Halstead , A. D. Miller , A. M. Schotthoefer , C. Stenoien , L. B. Johnson , and V. R. Beasley . 2015. “Predator Diversity, Intraguild Predation, and Indirect Effects Drive Parasite Transmission.” Proceedings of the National Academy USA 112: 3008–13. 10.1073/pnas.1415971112 PMC4364228 25713379

[eap2550-bib-0115] Rohr, J. R. , D. J. Civitello , F. W. Halliday , P. J. Hudson , K. D. Lafferty , C. L. Wood , and E. A. Mordecai . 2020. “Towards Common Ground in the Biodiversity–Disease Debate.” Nature Ecology and Evolution 4: 24–33. 31819238 10.1038/s41559-019-1060-6 PMC7224049

[eap2550-bib-0116] Rosà, R. , A. Pugliese , R. Norman , and P. J. Hudson . 2003. “Thresholds for Disease Persistence in Models for Tick‐Borne Infections Including Non‐viraemic Transmission, Extended Feeding and Tick Aggregation.” Journal of Theoretical Biology 224: 359–76. 12941594 10.1016/s0022-5193(03)00173-5

[eap2550-bib-0117] Rosenzweig, M. L. , and R. H. MacArthur . 1963. “Graphical Representation and Stability Conditions of Predator‐Prey Interactions.” American Naturalist 97: 209–23.

[eap2550-bib-0118] Rudolf, V. H. W. , and J. Antonovics . 2005. “Species Coexistence and Pathogens with Frequency‐Dependent Transmission.” American Naturalist 166: 112–8. 10.1086/430674 15937794

[eap2550-bib-0119] Ruyts, S. C. , E. Ampoorter , E. C. Coipan , L. Baeten , D. Heylen , H. Sprong , E. Matthysen , and K. Verheyen . 2016. “Diversifying Forest Communities May Change Lyme Disease Risk: Extra Dimension to the Dilution Effect in Europe.” Parasitology 143: 1310–9. 27173094 10.1017/S0031182016000688

[eap2550-bib-0120] Ruyts, S. C. , D. Landuyt , E. Ampoorter , D. Heylen , S. Ehrmann , E. C. Coipan , E. Matthysen , H. Sprong , and K. Verheyen . 2018. “Low Probability of a Dilution Effect for Lyme Borreliosis in Belgian Forests.” Ticks and Tick‐Borne Diseases 9: 1143–52. 29716838 10.1016/j.ttbdis.2018.04.016

[eap2550-bib-0121] Salkeld, D. J. , K. A. Padgett , and J. H. Jones . 2013. “A Meta‐Analysis Suggesting that the Relationship between Biodiversity and Risk of Zoonotic Pathogen Transmission Is Idiosyncratic.” Ecology Letters 16: 679–86. 23489376 10.1111/ele.12101 PMC7163739

[eap2550-bib-0122] Schmidt, K. A. , and R. S. Ostfeld . 2001. “Biodiversity and the Dilution Effect in Disease Ecology.” Ecology 82: 609–19.

[eap2550-bib-0123] Schwarz, C. J. , and A. N. Arnason . 1996. “A General Methodology for the Analysis of Capture‐Recapture Experiments in Open Populations.” Biometrics 52: 860–73.

[eap2550-bib-0124] Shoemaker, L. G. , E. Hayhurst , C. P. Weiss‐Lehman , A. T. Strauss , A. Porath‐Krause , E. T. Borer , E. W. Seabloom , and A. K. Shaw . 2019. “Pathogens Manipulate the Preference of Vectors, Slowing Disease Spread in a Multi‐Host System.” Ecology Letters 22: 1115–25. 31090159 10.1111/ele.13268

[eap2550-bib-0125] Siński, E. , A. Bajer , R. Welc , A. Pawełczyk , M. Ogrzewalska , and J. M. Behnke . 2006. “ *Babesia microti*: Prevalence in Wild Rodents and *Ixodes ricinus* Ticks from the Mazury Lakes District of North‐Eastern Poland.” International Journal of Medical Microbiology 296: 137–43. 16524774 10.1016/j.ijmm.2006.01.015

[eap2550-bib-0126] Smith, M. J. , A. White , X. Lambin , J. A. Sherratt , and M. Begon . 2006. “Delayed Density‐Dependent Season Length Alone Can Lead to Rodent Population Cycles.” American Naturalist 167: 695–704. 10.1086/503119 16671013

[eap2550-bib-0127] Sokolow, S. H. , N. Nova , K. M. Pepin , A. J. Peel , J. R. C. Pulliam , K. Manlove , P. C. Cross , et al. 2019. “Ecological Interventions to Prevent and Manage Zoonotic Pathogen Spillover.” Philosophical Transactions of the Royal Society B 374: 20180342. 10.1098/rstb.2018.0342 PMC6711299 31401951

[eap2550-bib-0128] Sprong, H. , S. Moonen , S. E. van Wieren , and T. R. Hofmeester . 2020. “Effects of Cattle Grazing on Ixodes Ricinus‐Borne Disease Risk in Forest Areas of The Netherlands.” Ticks and Tick‐Borne Diseases 11: 101355. 31837919 10.1016/j.ttbdis.2019.101355

[eap2550-bib-0129] Stenseth, N. C. , H. Viljugrein , W. Jȩdrzejewski , A. Mysterud , and Z. Pucek . 2002. “Population Dynamics of *Clethrionomys glareolus* and *Apodemus flavicollis*: Seasonal Components of Density Dependence and Density Independence.” Acta Theriologica 47: 39–67.

[eap2550-bib-0130] Strauss, A. T. , D. J. Civitello , C. E. Cáceres , and S. R. Hall . 2015. “Success, Failure and Ambiguity of the Dilution Effect among Competitors.” Ecology Letters 18: 916–26. 26119173 10.1111/ele.12468

[eap2550-bib-0131] Strauss, A. T. , M. S. Shocket , D. J. Civitello , J. L. Hite , R. M. Penczykowski , M. A. Duffy , C. E. Cáceres , and S. R. Hall . 2016. “Habitat, Predators, and Hosts Regulate Disease in Daphnia Through Direct and Indirect Pathways.” Ecological Monographs 86: 393–411.

[eap2550-bib-0132] Suzán, G. , E. Marcé , J. T. Giermakowski , J. N. Mills , G. Ceballos , R. S. Ostfeld , B. Armién , J. M. Pascale , and T. L. Yates . 2009. “Experimental Evidence for Reduced Rodent Diversity Causing Increased Hantavirus Prevalence.” PLoS One 4: e5461. 19421313 10.1371/journal.pone.0005461 PMC2673579

[eap2550-bib-0133] Telfer, S. , M. Bennett , K. Bown , R. Cavanagh , L. Crespin , S. Hazel , T. Jones , and M. Begon . 2002. “The Effects of Cowpox Virus on Survival in Natural Rodent Populations: Increases and Decreases.” Journal of Animal Ecology 71: 558–68.

[eap2550-bib-0134] Telfer, S. , R. Birtles , M. Bennett , X. Lambin , S. Paterson , and M. Begon . 2008. “Parasite Interactions in Natural Populations: Insights from Longitudinal Data.” Parasitology s135: 767–81. 10.1017/S0031182008000395 PMC2952918 18474121

[eap2550-bib-0135] Turchin, P. , and I. Hanski . 1997. “An Empirically Based Model for Latitudinal Gradient in Vole Population Dynamics.” American Naturalist 149: 842–74. 10.1086/286027 18811252

[eap2550-bib-0136] Venturino, E. 1994. “The Influence of Diseases on Lotka–Volterra Systems.” Rocky Mountain Journal of Mathematics 24: 381–402.

[eap2550-bib-0137] Venturino, E. 2001. “The Effect of Diseases on Competing Species.” Mathematical Biosciences 174: 111–31. 11730860 10.1016/s0025-5564(01)00081-5

[eap2550-bib-0138] Venturino, E. 2002. “Epidemics in Predator–Prey Models: Disease in the Predators.” Mathematical Medicine and Biology: A Journal of the IMA 19: 185–205. 12650334

[eap2550-bib-0139] Voordouw, M. J. , S. Lachish , and M. C. Dolan . 2015. “The Lyme Disease Pathogen Has No Effect on the Survival of Its Rodent Reservoir Host.” PLoS One 10: e0118265. 25688863 10.1371/journal.pone.0118265 PMC4331372

[eap2550-bib-0140] Welc‐Falęciak, R. , A. Bajer , J. M. Behnke , and E. Siński . 2008. “Effects of Host Diversity and the Community Composition of Hard Ticks (Ixodidae) on *Babesia Microti* Infection.” International Journal of Medical Microbiology 298: 235–42.

[eap2550-bib-0141] Werden, L. , I. K. Barker , J. Bowman , E. K. Gonzales , P. A. Leighton , L. R. Lindsay , and C. M. Jardine . 2014. “Geography, Deer, and Host Biodiversity Shape the Pattern of Lyme Disease Emergence in the Thousand Islands Archipelago of Ontario, Canada.” PLoS One 9: e85640. 24416435 10.1371/journal.pone.0085640 PMC3887107

[eap2550-bib-0142] White, G. C. , and K. P. Burnham . 1999. “Program MARK: Survival Estimation from Populations of Marked Animals.” Bird Study 46: S120–39.

[eap2550-bib-0143] Wojdak, J. M. , R. M. Edman , J. A. Wyderko , S. A. Zemmer , and L. K. Belden . 2014. “Host Density and Competency Determine the Effects of Host Diversity on Trematode Parasite Infection.” PLoS One 9: e105059. 25119568 10.1371/journal.pone.0105059 PMC4132046

[eap2550-bib-0144] Wood, C. L. , and K. D. Lafferty . 2013. “Biodiversity and Disease: A Synthesis of Ecological Perspectives on Lyme Disease Transmission.” Trends in Ecology and Evolution 28: 239–47. 23182683 10.1016/j.tree.2012.10.011

[eap2550-bib-0070] Wood, C. L. , K. D. Lafferty , G. DeLeo , H. S. Young , P. J. Hudson , and A. M. Kuris . 2014. “Does Biodiversity Protect Humans against Infectious Disease?” Ecology 95: 817–32. 24933803 10.1890/13-1041.1

[eap2550-bib-0145] Wright, D. H. , B. D. Patterson , G. M. Mikkelson , A. Cutler , and W. Atmar . 1997. “A Comparative Analysis of Nested Subset Patterns of Species Composition.” Oecologia 113: 1–20. 28307284 10.1007/s004420050348

[eap2550-bib-0146] Young, H. S. , R. Dirzo , K. M. Helgen , D. J. McCauley , S. A. Billeter , M. Y. Kosoy , L. M. Osikowicz , D. J. Salkeld , T. P. Young , and K. Dittmar . 2014. “Declines in Large Wildlife Increase Landscape‐Level Prevalence of Rodent‐Borne Disease in Africa.” Proceedings of the National Academy of Sciences USA 111: 7036–41. 10.1073/pnas.1404958111 PMC4024866 24778215

