# Supplementary material for: An eco‐epidemiological modeling approach to investigate dilution effect in two different tick‐borne pathosystems
Source: Ecol Appl. 2022 Mar 16;32(3):e2550. doi: 10.1002/eap.2550 (PMC9286340; doi:10.1002/eap.2550)
Supplement: Supplementary file 1 — Appendix S1 [file EAP-32-0-s002.pdf]

Supporting Information. Occhibove, F., Kenobi, K., Swain, M., Risley, C. An eco-epidemiological modeling approach to investigate dilution effect in two different tick-borne pathosystems. Ecological Applications

Appendix S1.

Modelling two tick-borne pathosystems

Appendix S1. Model equations (see Model structure and simulations section and Table 2 for complete notation and parameter values). b: bank vole; w: wood mouse; j: shrew (competitor species); p: weasel (specialist predator); K: carrying capacity; l: larva; n: nymph; a: adult tick.

$$\frac{dN_w}{dt} = r_w N_w \left(1 - \frac{(N_w - c_{wb}N_b - c_{wj}N_j)}{K_w}\right) - \frac{(gN_w^2)}{(N_w^2 + h^2)} - (\alpha_w N_p N_w)/\left(\Delta_w + N_w + \frac{\Delta_w}{\Delta_b}N_b + \frac{\Delta_w}{\Delta_j}N_j\right) \quad \text{Eq. S1}$$

$$\frac{dN_b}{dt} = r_b N_b \left(1 - \frac{(N_b - c_{bw}N_w - c_{bj}N_j)}{K_b}\right) - \frac{(gN_b^2)}{(N_b^2 + h^2)} - (\alpha_b N_p N_b)/\left(\Delta_b + N_b + \frac{\Delta_b}{\Delta_w}N_w + \frac{\Delta_b}{\Delta_j}N_j\right) \quad \text{Eq. S2}$$

$$\frac{dN_j}{dt} = (v_j - \rho_j)N_j \left(1 - \frac{(N_j - c_{jw}N_w - c_{jb}N_b)}{K_j}\right) - \frac{\left(gN_j^2\right)}{\left(N_j^2 + h^2\right)} - (\alpha_j N_p N_j)/\left(\Delta_j + N_j + \frac{\Delta_j}{\Delta_w}N_w + \frac{\Delta_j}{\Delta_b}N_b\right) \quad \text{Eq. S3}$$

$$\frac{dN_p}{dt} = (v_p - \rho_p)N_p \left(1 - q\,N_p/\left(\Delta_w + N_w + \frac{\Delta_w}{\Delta_b}N_b + \frac{\Delta_w}{\Delta_j}N_j\right)\right) \quad \text{Eq. S4}$$

$$\frac{dl}{dt} = (\beta_7 d_1 N_w a + \beta_7 d_1 N_b a + \beta_8 d_2 N_j a + \beta_9 d_3 N_p a + \beta_9 d_4 N_d a)(num_{egg} - s_v N_v) - \rho_v l$$

$$-(\beta_1 N_w l + \beta_1 N_b l + \beta_2 N_j l + \beta_3 N_p l + \beta_3 N_d l)(1 + 1/k) \quad \text{Eq. S5}$$

$$\frac{dn}{dt} = (\beta_1 d_1 N_w l + \beta_1 d_1 N_b l + \beta_2 d_2 N_j l + \beta_3 d_3 N_p l + \beta_3 d_4 N_d l) - \rho_v n$$

$$-(\beta_4 N_w n + \beta_4 N_b n + \beta_5 N_j n + \beta_6 N_p n + \beta_6 N_d n)(1 + 1/k) \quad \text{Eq. S6}$$

$$\frac{da}{dt} = (\beta_4 d_1 N_w n + \beta_4 d_1 N_b n + \beta_5 d_2 N_j n + \beta_6 d_3 N_p n + \beta_6 d_4 N_d n) - \rho_v a$$

$$-(\beta_7 N_w a + \beta_7 N_b a + \beta_8 N_j a + \beta_9 N_p a + \beta_9 N_d a)(1 + 1/k) \quad \text{Eq. S7}$$

$$\frac{dS_w}{dt} = r_w N_w \left(1 - \frac{(N_w - c_{wb}N_b - c_{wj}N_j)}{K_w}\right) - \beta_4 \tau_v S_w I_n - \beta_7 \tau_v S_w I_a - \frac{(gS_w^2)}{(S_w^2 + h^2)}$$

$$-(\alpha_w N_p S_w)/\left(\Delta_w + N_w + \frac{\Delta_w}{\Delta_b}N_b + \frac{\Delta_w}{\Delta_j}N_j\right) \quad \text{Eq. S8}$$

$$\frac{dI_w}{dt} = \beta_4 \tau_v S_w I_n - \beta_7 \tau_v S_w I_a - \sigma_w I_w - \frac{(gI_w^2)}{(I_w^2 + h^2)}$$

$$-(\alpha_w N_p I_w)/\left(\Delta_w + N_w + \frac{\Delta_w}{\Delta_b}N_b + \frac{\Delta_w}{\Delta_j}N_j\right) \quad \text{Eq. S9}$$

$$\frac{dR_w}{dt} = \sigma_w I_w - \frac{(gR_w^2)}{(R_w^2+h^2)} - (\alpha_w N_p R_w)/\left(\Delta_w + N_w + \frac{\Delta_w}{\Delta_b} N_b + \frac{\Delta_w}{\Delta_j} N_j\right) \qquad \text{Eq. S10}$$

$$\frac{dS_b}{dt} = r_b N_b \left(1 - \frac{(N_b - c_{bw} N_w - c_{bj} N_j)}{K_b}\right) - \beta_4 \tau_v S_b I_n - \beta_7 \tau_v S_b I_a - \frac{(gS_b^2)}{(S_b^2 + h^2)}$$

$$-(\alpha_b N_p S_b)/\left(\Delta_b + N_b + \frac{\Delta_b}{\Delta_w} N_w + \frac{\Delta_b}{\Delta_j} N_j\right) \qquad \text{Eq. S11}$$

$$\frac{dI_b}{dt} = \beta_4 \tau_v S_b I_n - \beta_7 \tau_v S_b I_a - \sigma_b I_b - \frac{(gI_b^2)}{(I_b^2+h^2)} - (\alpha_b N_p I_b)/\left(\Delta_b + N_b + \frac{\Delta_b}{\Delta_w} N_w + \frac{\Delta_b}{\Delta_j} N_j\right) \qquad \text{Eq. S12}$$

$$\frac{dR_b}{dt} = \sigma_b I_b - \frac{(gR_b^2)}{(R_b^2+h^2)} - (\alpha_b N_p R_b)/\left(\Delta_b + N_b + \frac{\Delta_b}{\Delta_w} N_w + \frac{\Delta_b}{\Delta_j} N_j\right) \qquad \text{Eq. S13}$$

$$\frac{dl}{dt} = (\beta_7 d_1 N_w + \beta_7 d_1 N_b + \beta_8 d_2 N_j + \beta_9 d_3 N_p + \beta_9 d_4 N_d)(S_a + I_a)(num_{egg} - s_v N_v) - \rho_v l - (\beta_1 N_w l + \beta_1 N_b l +$$

$$\beta_2 N_j l + \beta_3 N_p l + \beta_3 N_d l)(1 + 1/k) \qquad \text{Eq. S14}$$

$$\frac{dI_n}{dt} = (\beta_1 d_1 I_w \tau_w l + \beta_1 d_1 I_b \tau_b l)(1 + 1/k) - \rho_v I_n - (\beta_4 N_w I_n + \beta_4 N_b I_n + \beta_5 N_j I_n + \beta_6 N_p I_n + \beta_6 N_d I_n)(1 + 1/k)$$

$$\text{Eq. S15}$$

$$\frac{dS_n}{dt} = (\beta_1 d_1 (S_w + R_w) l + \beta_1 d_1 (S_b + R_b) l + \beta_2 d_2 N_j l + \beta_3 d_3 N_p l + \beta_3 d_4 N_d l) - \rho_v S_n$$

$$-(\beta_4 N_w S_n + \beta_4 N_b S_n + \beta_5 N_j S_n + \beta_6 N_p S_n + \beta_6 N_d S_n) \qquad \text{Eq. S16}$$

$$\frac{dI_a}{dt} = (\beta_4 d_1 N_w I_n + \beta_4 d_1 N_b I_n + \beta_5 d_2 N_j I_n + \beta_6 d_3 N_p I_n + \beta_6 d_4 N_d I_n) + (\beta_4 d_1 I_w \tau_w S_n + \beta_4 d_1 I_b \tau_b S_n)(1 + 1/k)$$

$$-\rho_v I_a - (\beta_7 N_w I_a + \beta_7 N_b I_a + \beta_8 N_j I_a + \beta_9 N_p I_a + \beta_9 N_d I_a)(1 + 1/k) \qquad \text{Eq. S17}$$

$$\frac{dS_a}{dt} = (\beta_4 d_1 (S_w + R_w) S_n + \beta_4 d_1 (S_b + R_b) S_n + \beta_5 d_2 N_j S_n + \beta_6 d_3 N_p S_n + \beta_6 d_4 N_d S_n) - \rho_{v4} S_a - (\beta_7 N_w S_a +$$

$$\beta_7 N_b S_a + \beta_8 N_j S_a + \beta_9 N_p S_a + \beta_9 N_d S_a) \qquad \text{Eq. S18}$$
