# Supplementary material for: An eco‐epidemiological modeling approach to investigate dilution effect in two different tick‐borne pathosystems
Source: Ecol Appl. 2022 Mar 16;32(3):e2550. doi: 10.1002/eap.2550 (PMC9286340; doi:10.1002/eap.2550)
Supplement: Supplementary file 2 — Appendix S2 [file EAP-32-0-s004.pdf]

Supporting Information. Occhibove, F., Kenobi, K., Swain, M., Risley, C. An eco-epidemiological modeling approach to investigate dilution effect in two different tick-borne pathosystems. Ecological Applications

Appendix S2

Modelling two tick-borne pathosystems

Appendix S2. Parameters estimation methodology

Parameters estimated from rodent live-trapping in this study included wood mouse and bank vole growth rates (*r*) as described in the Ground dwelling rodent populations survey section of the Methods. In addition, rodent species body mass allowed the allometric estimation of the carrying capacity (*K*) using the formula: $K= 16.2w^{-0.70}$ where *w* is the mean body mass in grams (De Leo and Dobson 1996, Bolzoni et al. 2008). Rodents' body mass values were the average of adults and sub-adults (this study) of each species.

Adult body mass values from PanTHERIA database (Jones et al. 2009) were used to estimate carrying capacity (*K*) (same formula above), birth (*ν*), and death rate (*ρ*) of common shrew and least weasel using the following allometric formulae: $\nu=w^{-0.25}$, $\rho=0.4w^{-0.25}$.

The competition factor (*c*) was computed algebraically, ranging from 0 (competition absent) to the maximum value for the competing species to co-exist, K_j/K_i in the case of species *i* competing over species *j*. Predation parameters were estimated with methods in Turchin and Hanski (1997), using empirical data from this study or data specific to the UK. These were estimated separately for rodents and shrews, as the latter have a significantly lower body mass and are secondary prey items (Henttonen et al. 1989).

Tick aggregation parameter (*k*) was calculated from empirical data: it represents the dispersion parameter of the negative binomial distribution fitting field data of ticks collected on rodent hosts (pooled rodent species: k = 0.18, μ = 0.23, D = 264.14, df = 543; analysis performed with the function glm.nb, link = log, package MASS, R Core Team, 2016).

Literature Cited

Bolzoni, L., G. a. De Leo, M. Gatto, and A. P. Dobson. 2008. Body-size scaling in an SEI model of wildlife diseases. Theoretical Population Biology 73:374–382.

De Leo, G. A., and P. A. Dobson. 1996. Allometry and simple epidemic models for microparasites. Nature 379:720–722.

Henttonen, H., V. Haukisalmi, and A. Kaikusalo. 1989. Long-term population dynamics of the common shrew *Sorex araneus* in Finland. Annales Zoologici Fennici 26:349–355.

Jones, K. E., J. Bielby, M. Cardillo, S. A. Fritz, J. O'Dell, C. D. L. Orme, K. Safi, W. Sechrest, E. H. Boakes, C. Carbone, C. Connolly, M. J. Cutts, J. K. Foster, R. Grenyer, M. Habib, C. A. Plaster, S. A. Price, E. A. Rigby, J. Rist, A. Teacher, O. R. P. Bininda-Emonds, J. L. Gittleman, G. M. Mace, and A. Purvis. 2009. PanTHERIA: a species-level database of life history, ecology, and geography of extant and recently extinct mammals. Ecology 90: 2648-2648.

R Core Team. 2016. R: A language and environment for statistical computing. R-3.2.5 for Windows (32/64 bit). R Foundation for Statistical Computing, Vienna, Austria. https://www.R-project.org/.

Turchin, P., and I. Hanski. 1997. An Empirically Based Model for Latitudinal Gradient in Vole Population Dynamics. The American Naturalist 149:842–874.
