# Supplementary material for: An eco‐epidemiological modeling approach to investigate dilution effect in two different tick‐borne pathosystems
Source: Ecol Appl. 2022 Mar 16;32(3):e2550. doi: 10.1002/eap.2550 (PMC9286340; doi:10.1002/eap.2550)
Supplement: Supplementary file 3 — Appendix S3 [file EAP-32-0-s003.pdf]

Supporting Information. Occhibove, F., Kenobi, K., Swain, M., Risley, C. An eco-epidemiological modeling approach to investigate dilution effect in two different tick-borne pathosystems. Ecological Applications

Appendix S3

Modelling two tick-borne pathosystems

Appendix S3. Additional tables and figures.

Table S1. Mean growth rate values by season and rodent species. Sample size (referring to the number of seasons) in brackets. Breeding: March to October. Non-breeding: November to February. St.Er: standard error.

**Species**	**Season**	**Mean**	**St.Er**
Bank vole	Breeding	0.444 (3)	0.163
Wood mouse	Breeding	0.413 (5)	0.151
Bank vole	Non-breeding	-0.196 (6)	0.141
Wood mouse	Non-breeding	-0.526 (8)	0.207

Figure S1. Effect of inter-specific competition and predation on rodent populations across the 20 years simulation timescale. Plot shows the change in total number of bank voles and wood mice across different scenarios. Solid line: bank vole; dotted line: wood mouse. Black: scenario 1; purple: scenario 2 (effect of competition among the two rodent species); teal: scenario 3 (effect of shrew competition with rodents); yellow: scenario 4 (effect of generalist and specialist predation). Scenario 5 not displayed as, by model assumption, the population of ungulates did not affect other species population. Parameter values listed in Table 2.

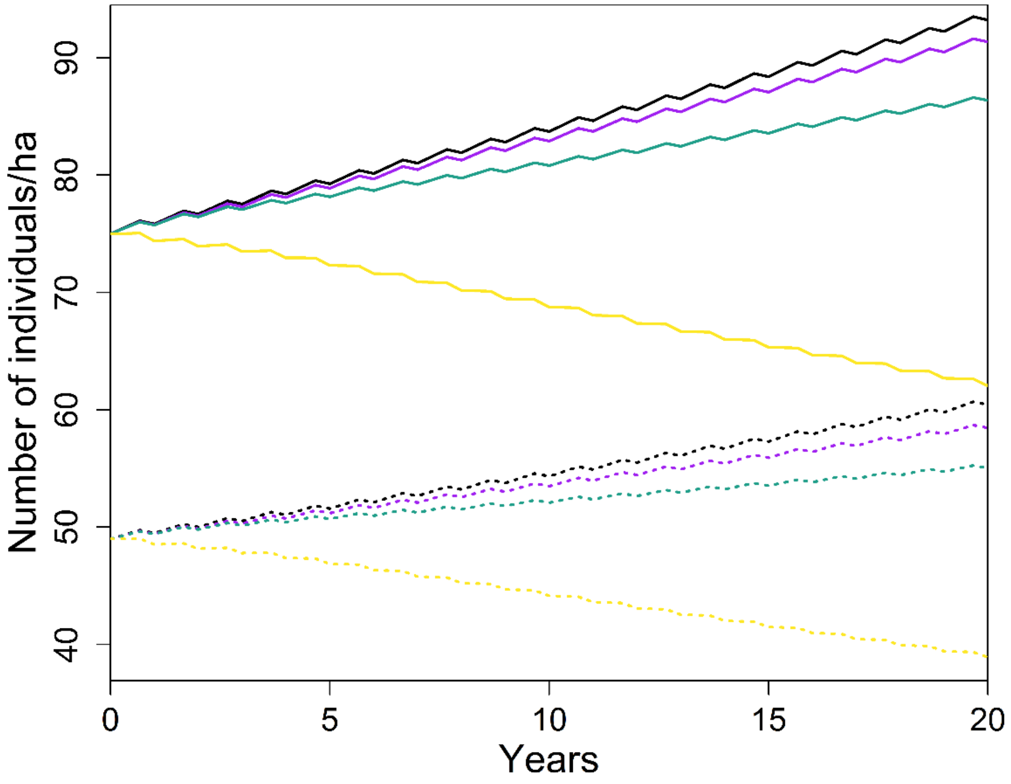

Figure S2. Host-vector dynamics in absence of pathogens: comparison between *Ixodes ricinus* (a, b, c, d) and *I. trianguliceps* (e, f, g, h) systems. Log transformed number of individual larvae (dashed line), nymphs (solid line), and adults (dotted line) across different community assemblages (Scenarios in Table 1). Parameter values listed in Table 2.

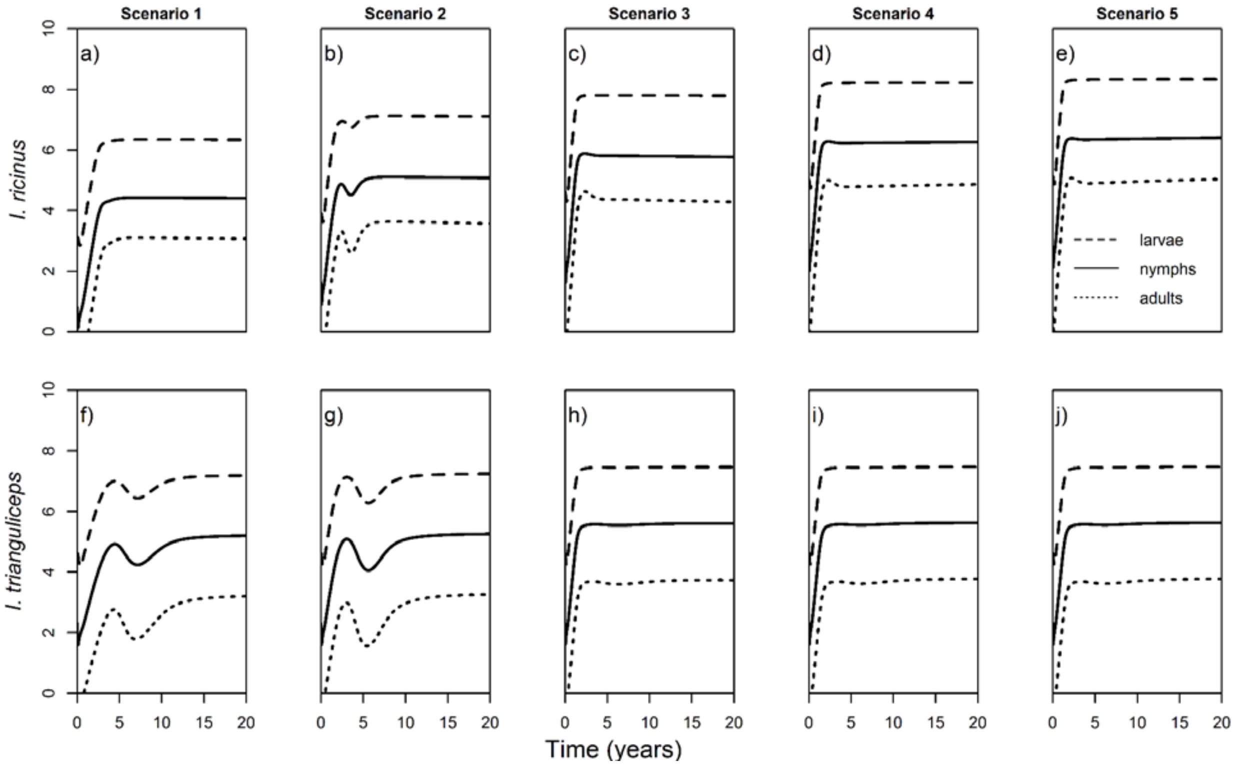

Fig. S3. PRCC values for the models simulating the two pathosystems, using as response functions (a) density of infectious nymphs (DIN), and (b) number of infectious hosts. Parameter values (and ranges) listed in Table 2.

a)

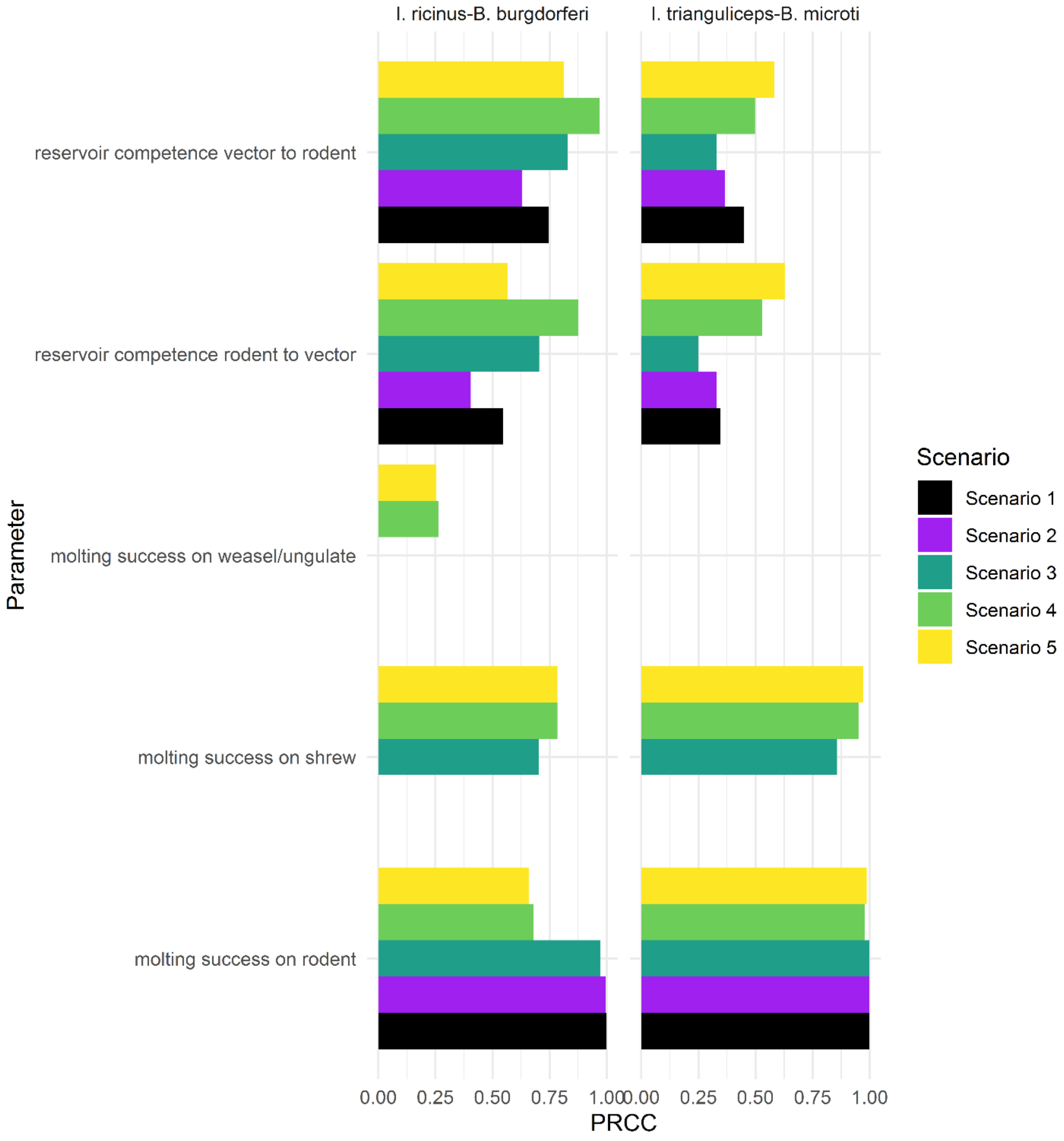

b)

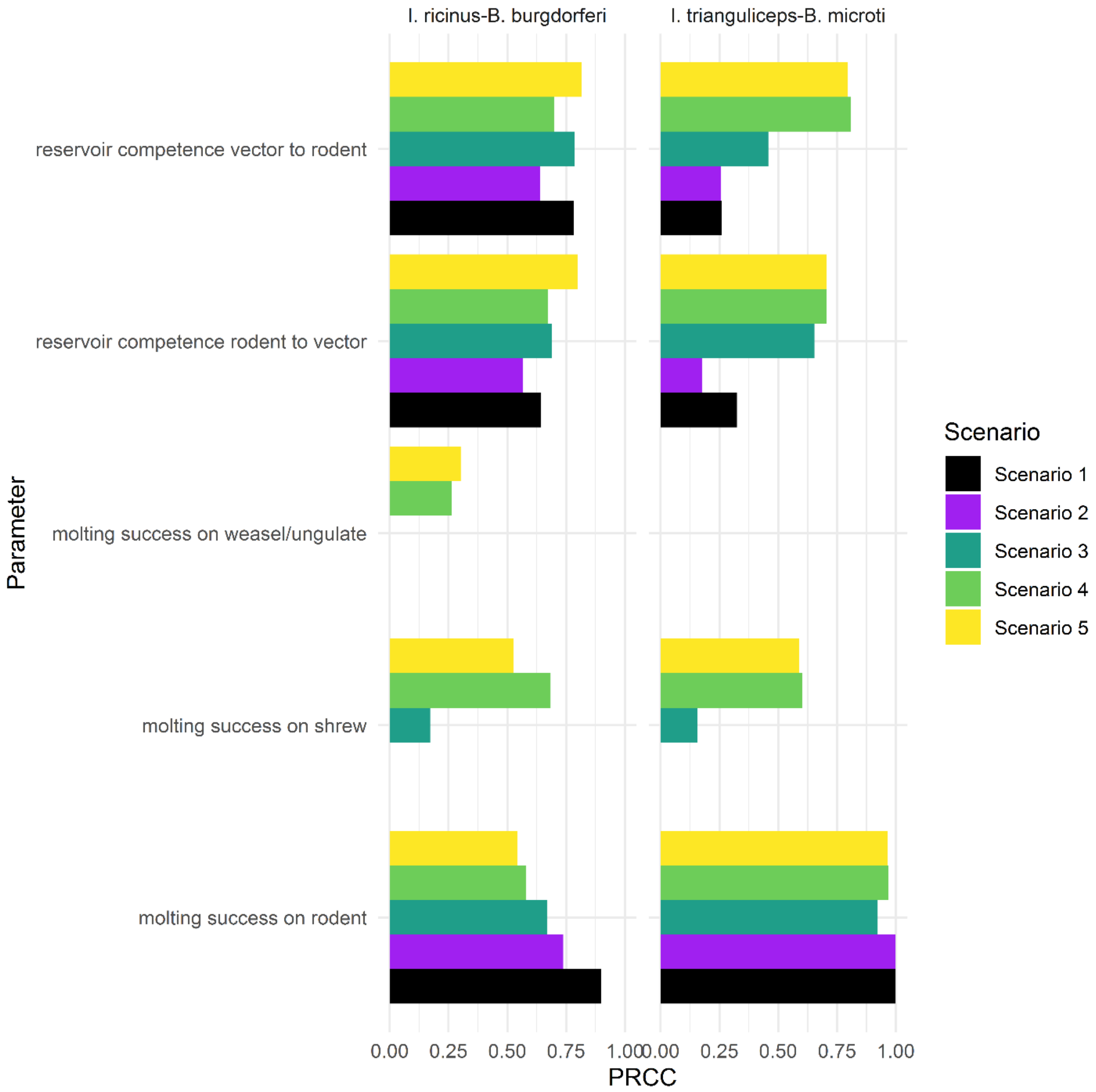
I. ricinus-B. burgdorferi
I. trianguliceps-B. microti
reservoir competence vector to rodent
reservoir competence rodent to vector
molting success on weasel/ungulate
molting success on shrew
molting success on rodent
Parameter
0.00
0.25
0.50
0.75
1.00
PRCC
Scenario
Scenario 1
Scenario 2
Scenario 3
Scenario 4
Scenario 5
