# Supplementary material for: An eco‐epidemiological modeling approach to investigate dilution effect in two different tick‐borne pathosystems
Source: Ecol Appl. 2022 Mar 16;32(3):e2550. doi: 10.1002/eap.2550 (PMC9286340; doi:10.1002/eap.2550)
Supplement: Supplementary file 4 — Data S1 [file EAP-32-0-s001.zip › MetadataS1.pdf]

Occhibove, F., Kenobi, K., Swain, M., Risley, C. 2022. An eco-epidemiological modeling approach to investigate dilution effect in two different tick-borne pathosystems. *Ecological Applications.*

Data S1

Tick_borne_model.R

Authors

Flavia Occhibove
IBERS, Aberystwyth University
SY23 3EB Aberystwyth, UK
UK Centre for Ecology & Hydrology
OX10 8BB, Wallingford, UK
flaocc@ceh.ac.uk

Kenobi Kim
Department of Mathematics, Aberystwyth University
SY23 3BZ Aberystwyth, UK
kik10@aber.ac.uk

Claire Risley
IBERS, Aberystwyth University
SY23 3EB Aberystwyth, UK
Clr25@aber.ac.uk

File list

`Tick_borne_model.R`

Description

`Tick_borne_model.R` - Code including the model function to reproduce all the results included in the paper. To simulate the different scenarios and or conditions described in the paper parameter values and starting conditions need to be varied accordingly. All these are available in Table 2 of the main text.
